# Autophagy controls the induction and developmental decline of NMDAR-LTD through endocytic recycling

**DOI:** 10.1038/s41467-020-16794-5

**Published:** 2020-06-12

**Authors:** Hongmei Shen, Huiwen Zhu, Debabrata Panja, Qinhua Gu, Zheng Li

**Affiliations:** 1 Section on Synapse Development and Plasticity, National Institute of Mental Health, National Institutes of Health, Bethesda, MD 20892 USA; 2 Present Address: Key Laboratory of Neuroregeneration of Jiangsu and Ministry of Education & Co-innovation Center of Neuroregeneration, Nantong University, Nantong, 226001 China; 3 Present Address: Nantong Brain Hospital & Mental Health Center Affiliated to Nantong University, Nantong University, Nantong, 226005 China

**Keywords:** Macroautophagy, Endocytosis, Synaptic plasticity

## Abstract

NMDA receptor-dependent long-term depression (NMDAR-LTD) is a long-lasting form of synaptic plasticity. Its expression is mediated by the removal of AMPA receptors from postsynaptic membranes. Under basal conditions, endocytosed AMPA receptors are rapidly recycled back to the plasma membrane. In NMDAR-LTD, however, they are diverted to late endosomes for degradation. The mechanism for this switch is largely unclear. Additionally, the inducibility of NMDAR-LTD is greatly reduced in adulthood. The underlying mechanism and physiological significance of this phenomenon are elusive. Here, we report that autophagy inhibition is essential for the induction and developmental dampening of NMDAR-LTD. Autophagy is inhibited during NMDAR-LTD to decrease endocytic recycling. Autophagy inhibition is both necessary and sufficient for LTD induction. In adulthood, autophagy is up-regulated to make LTD induction harder, thereby preventing the adverse effect of excessive LTD on memory consolidation. These findings reveal the unrecognized functions of autophagy in synaptic plasticity, endocytic recycling, and memory.

## Abstract (web summary)

NMDA receptor-dependent long-term depression (NMDAR-LTD) is a form of synaptic plasticity mediated by reduced recycling of AMPA receptors to the plasma membrane. Here the authors show that autophagy is a regulator of this endocytic recycling and autophagy upregulation dampens NMDAR-LTD in adulthood.

## Introduction

Synaptic plasticity, the ability of synapses to change in strength, is a cellular mechanism underlying information processing and storage in the brain. NMDA receptor-dependent long-term depression (NMDAR-LTD) is essential for contextual fear memory, spatial memory, contextual habituation, spatial reversal learning, behavioral flexibility, novelty acquisition, and social recognition^1–9^. Although NMDAR-LTD is can be induced by learning in adult animals, it is more readily induced in the first three postnatal weeks^10–13^. Little is known about how NMDAR-LTD is down-regulated in adults and the physiological significance of this phenomenon.

NMDAR-LTD is triggered by NMDA receptor activation which causes Ca^2+^ increase, activation of Ca^2+^-dependent protein phosphatases, and subsequent GluA1 dephosphorylation and activation of the mitochondria-caspase pathway^14–16^. These processes drive the removal of synaptic AMPA receptors. AMPA receptor removal begins with clathrin-dependent endocytosis, followed by sorting into either recycling endosomes (for reinsertion into the plasma membrane) or late endosomes (for degradation in lysosomes)^17^. While AMPA receptors rapidly recycle back to plasma membranes under basal conditions, they are diverted from recycling to late endosomes after NMDA receptor activation^18,19^. The mechanism for suppressing AMPA receptor recycling during NMDAR-LTD is largely unclear.

Macroautophagy (autophagy hereinafter) is a process through which proteins and organelles are delivered to lysosomes for degradation^20,21^. The best-studied function of autophagy is cellular homeostasis^22,23^. Neuronal autophagy also contributes to dopamine release, presynaptic assembly, axonal outgrowth, and dendritic spine pruning^24–28^. A role of autophagy in LTD has been hypothesized as NMDA receptor activation causes a delayed increase in autophagic flux in cultured hippocampal neurons^29^. This hypothesis, however, is incompatible with the finding that autophagy inhibitors have no effect on hippocampal LTD in mice until they are aged and accumulate Tau oligomers^30^. The role of autophagy in LTD remains elusive.

Here, we show that autophagic flux in CA1 neurons is transiently decreased during the induction phase of NMDAR-LTD. This autophagy inhibition causes a reduction of endocytic recycling and is required for AMPA receptor internalization and synaptic depression. Moreover, autophagy is upregulated during development to decrease the inducibility of NMDAR-LTD in adults to ensure proper consolidation of associative fear memory.

## Results

### Autophagy is inhibited in LTD

To test whether autophagy is involved in synaptic plasticity, we analyzed autophagy during LTP and LTD using two methods: time-lapse imaging and immunoblotting for LC3 and p62. The cleaved and phosphatidylethanolamine conjugated form of LC3 (LC3-II) is incorporated into autophagosomes and remains on mature autophagosomes^31–33^. LC3-II in combination with p62 (a protein selectively degraded by autophagy) indicates autophagic flux^34–38^.

**Fig. 1:**
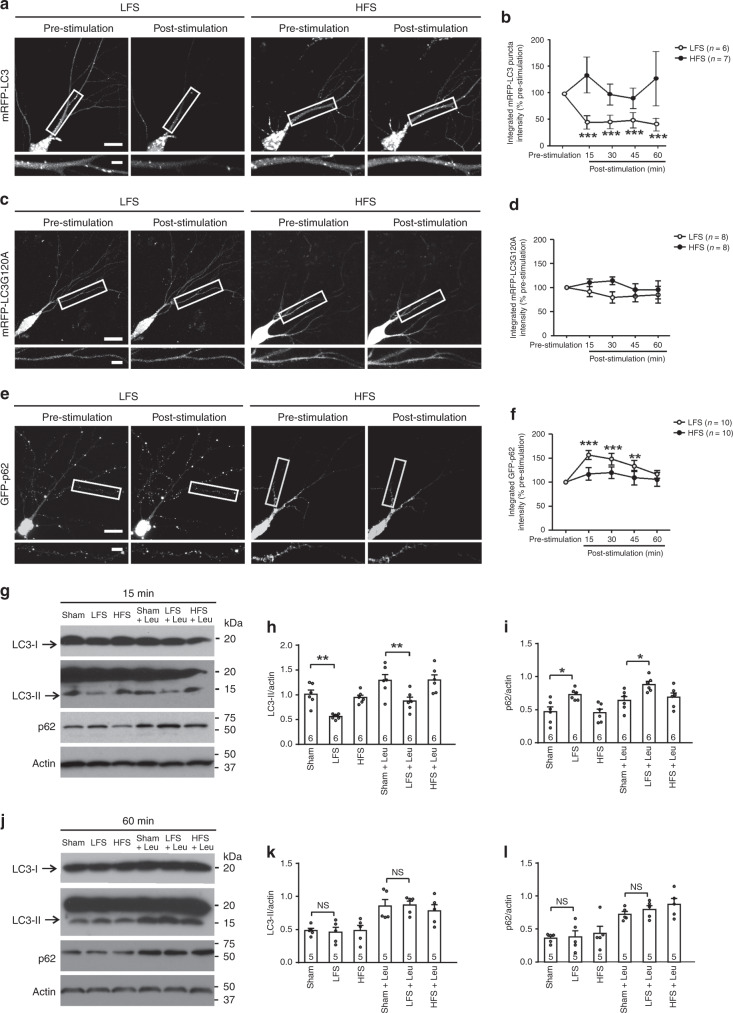
Autophagy is inhibited by low-frequency stimulation that induces LTD. **a**, **c**, **e** Representative images of the same dendrites of CA1 neurons in cultured hippocampal slices before and after stimulation at the Schaffer collateral pathway with low-frequency stimulation (LFS) or high-frequency stimulation (HFS); scale bars, 20 μm in the upper images and 5 μm in the lower images. **b**, **d**, **f** Quantification of integrated fluorescence intensity for mRFP-LC3 puncta in **a**, mRFP-LC3G120A in **c**, and GFP-p62 in **e**; one-way RM ANOVA or Kruskal–Wallis one-way RM ANOVA on Ranks was used to compare different time points within the HFS or LFS group (**b**: *p* = 9.4 × 10^−5^ for LFS, *p* = 0.704 for HFS; **d**: *p* = 0.454 for LFS, *p* = 0.570 for HFS; **f**: *p* = 2.35 × 10^−7^ for LFS, *p* = 0.399 for HFS); Holm–Sidak was used to identify the post-stimulation time points significantly different from the pre-stimulation level in the LFS group (indicated by asterisks); *n* indicates the number of slices, and one slice per animal was used. **g**, **j** Representative blots of CA1 tissues taken from acute hippocampal slices stimulated with LFS or HFS at the Schaffer collateral pathway. **h**, **i** Quantification of LC3-II and p62 for **g**. **k**, **l** Quantification of LC3-II and p62 for **j**. In **g**–**l**, the number in the bars indicates the number of animals for each condition; one-way ANOVA was used to compare across groups (**h**: *p* = 5 × 10^−6^; **i**: *p* = 0.00005; **k**: *p* = 0.000423; **l**: *p* = 5.30 × 10^−5^; groups significantly different from the sham group were identified with Student–Newman–Keuls test and marked by asterisks. No adjustments were made for multiple comparisons Data are presented as mean ± SEM; *p < 0.05, **p < 0.01, ***p < 0.001. Source data are provided as a Source Data file.

For imaging, cultured hippocampal slices (7 days in vitro or DIV 7) were biolistically transfected to express monomeric red fluorescent protein-tagged LC3 (mRFP-LC3), mRFP-LC3G120A (lipidation-defective LC3 mutant that cannot be incorporated into autophagosomes, therefore exhibits a diffuse pattern of cellular expression), or GFP tagged p62 (GFP-p62). At 3–5 days after transfection, we stimulated the Schaffer collateral pathway (SC) with high-frequency stimulation (HFS; 100 Hz, 2 trains of 100 pulses with an intertrain interval of 15 s) for LTP induction or low-frequency stimulation (LFS; 1 Hz, 900 pulses) for LTD induction. LFS decreased mRFP-LC3 puncta at all time points measured after stimulation and had no effect on mRFP-LC3G120A (Fig. 1a–d). LFS also increased the integrated intensity of p62 at 15 min and 30 min after stimulation (Fig. 1e, f). HFS had no significant effect on mRFP-LC3 puncta, mRFP-LC3G120A or p62 (Fig. 1a–f). These results indicate that LFS causes a transient inhibition of autophagy.

For immunoblotting, acute hippocampal slices from 16–19-day-old mice were stimulated at SC with LFS or HFS. The CA1 region near the stimulating electrode was removed for immunoblotting. While LC3-II decreased and p62 increased at 15 min post-stimulation (Fig. 1g–i), they returned to pre-stimulation levels by 60 min post-stimulation (Fig. 1j–l). The hippocampal tissue used for immunoblotting contained neurites and somas, while only dendrites of pyramidal neurons were measured by imaging. This difference may account for the lack of LC3 changes in tissue lysates at 60 min poststimulation. The alteration of LC3-II and p62 at 15 min and recovery by 60 min poststimulation was preserved in the presence of leupeptin (Fig. 1g–l), a non-lysosomotropic autophagy inhibitor, indicating that the autophagy inhibition is transient. Leupeptin increased both LC3-II and p62 in slices because of the inhibition of autophagic flux (Supplementary Fig. 1A–C). It is noted that because LC3-II is an intermediary protein in autophagy, autophagy impairment can cause either increase or decrease of LC3. HFS had no significant effect on LC3-II or p62 regardless of leupeptin treatment (Fig. 1g–l).

Taken together, these results show that autophagy is inhibited in LTD but not in LTP.

### Autophagy inhibition is required for LTD induction

**Fig. 2:**
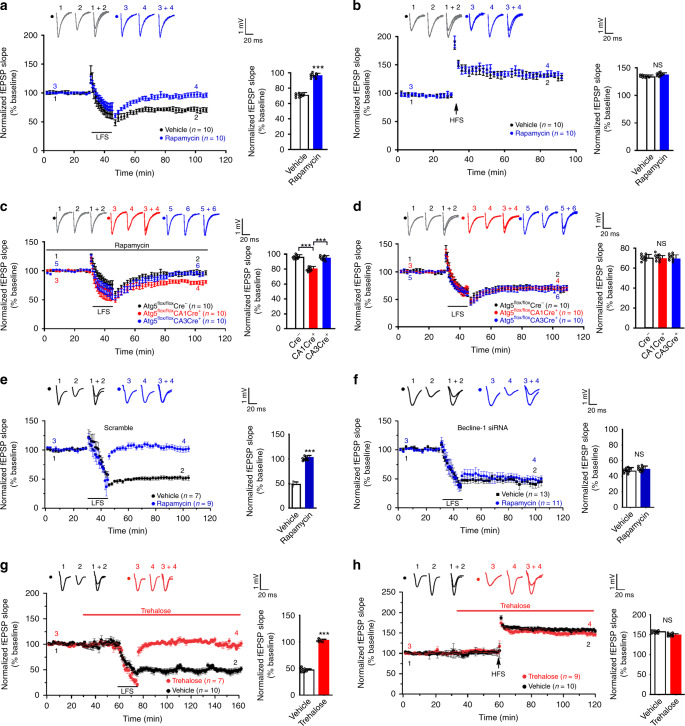
Autophagy inhibition is required for LTD Induction. Acute hippocampal slices (**a**–**d**, **g**, **h**) or cultured hippocampal slices (**e**, **f**) were stimulated at the Schaffer collateral pathway and recorded in the CA1 region for field excitatory postsynaptic potentials (fEPSPs). **a**, **b** Wild-type acute slices were perfused with vehicle or rapamycin (1 μM) for 30 min before LFS (for LTD induction) or HFS (for LTP induction) and throughout the recording period; quantification on the right shows the average slope of fEPSPs recorded at 50–60 min after LFS or HFS normalized to the pre-stimulation baseline; two-tailed Student’s *t* test was used for statistical analysis. **c**, **d** Acute slices taken from Atg5^flox/flox^Cre^+^ and Atg5^flox/flox^Cre^−^ mice were stimulated with LFS; slices in **c** were perfused with rapamycin (1 μM) for 30 min before LFS and throughout the recording period; quantification on the right shows the average slope of fEPSPs recorded at 50–60 min after LFS normalized to the pre-stimulation baseline; one-way ANOVA was used for comparison across groups; Bonferroni test was used to identify groups significantly different from the Atg5^flox/flox^Cre^−^ group in **c**; no adjustments were made for multiple comparisons. **e**, **f** Wild-type, cultured hippocampal slices were perfused with vehicle or rapamycin (1 μM) for 30 min before LFS and throughout the recording period; quantification on the right shows the average slope of fEPSPs recorded at 50–60 min after LFS normalized to the pre-stimulation baseline; two-tailed Student’s *t* test was used for statistical analysis. **g**, **h** Wild-type, acute slices were perfused with vehicle or trehalose (20 mM) for 30 min before LFS (for LTD induction in **g**) or HFS (for LTP induction in **h**) and throughout the recording period; quantification on the right shows the average slope of fEPSPs recorded at 50–60 min after LFS or HFS normalized to the pre-stimulation baseline; two-tailed Student’s *t* test was used for statistical analysis. Data are presented as mean ± SEM; *n* indicates the number of slices (one slice from each animal); ***p < 0.001. LFS: low-frequency stimulation. HFS high-frequency stimulation. Source data are provided as a Source Data file.

To examine the effect of autophagy inhibition on LTD, we activated autophagy in acute hippocampal slices with rapamycin which suppresses the initiation of autophagosome formation^19,39,40^. Rapamycin increased LC3-II and decreased p62 in hippocampal slices (Supplementary Fig. 1A–C). Field excitatory postsynaptic potentials (fEPSPs) were recorded in the CA1 region by stimulating SC. Rapamycin blocked LTD (fEPSPs at 50–60 min after LFS normalized to the pre-stimulation baseline: 70.54 ± 3.61% in vehicle-treated and 95.71 ± 3.62% in rapamycin-treated slices; two-tailed Student’s *t* test, *p* = 6.8 × 10^−20^; Fig. 2a) and had no effect on LTP (fEPSPs at 50–60 min after HFS normalized to the pre-stimulation baseline: 133.33 ± 3.59% in vehicle-treated and 136.46 ± 4.23% in rapamycin-treated slices; two-tailed Student’s *t* test, *p* = 0.15; Fig. 2b). The input-output relationship and paired-pulse ratio were intact in rapamycin-treated slices (Supplementary Fig. 1D, E).

To test whether rapamycin blocks LTD via autophagy and whether at pre- or post-synaptic sites, we generated CA1-specific and CA3-specific Atg5 knockout mice by crossing floxed Atg5 mice with mice expressing Cre selectively in the CA1 or CA3 region^41,42^. In P19 mice, the reduction of Atg5 in the CA1 area of *Atg5*^*flox/flox*^*CA1Cre*^*+*^ mice and in the CA3 area of *Atg5*^*flox/flox*^*CA3Cre*^*+*^ mice were comparable (Supplementary Fig. 1F–H).

Rapamycin blocked LTD in CA1-Cre^−^ and CA3-Cre^+^ slices, but not in CA1-Cre^+^ slices [fEPSPs at 50–60 min after LFS normalized to the pre-stimulation baseline: 95.60 ± 0.46% in Cre^−^ slices; 95.16 ± 0.35% in CA3-Cre^+^ slices; 80.52 ± 0.49% in CA1-Cre^+^ slices; one-way ANOVA (*p* = 1.3 × 10^−20^) and post hoc Bonferroni (*p* = 1.7 × 10^−19^ for CA1-Cre^+^ vs. Cre^−^; *p* = 3.7 × 10^−19^ for CA3-Cre^+^ vs. CA1-Cre^+^) were used for statistical analysis; Fig. 2c]. LTP, the input-output relationship, and paired-pulse ratio were intact in CA1-specific Atg5 knockout mice (Fig. 2d, Supplementary Fig. 1I, J). Since CA1 neurons are postsynaptic while CA3 neurons are presynaptic in SC synapses, these results indicate that rapamycin inhibits LTD through postsynaptic autophagy.

Moreover, we inhibited autophagy by using Beclin-1 siRNAs. The efficacy and specificity of Beclin-1 siRNAs were tested in primary hippocampal neurons (Supplementary Fig. 1K–N). Lentivirus expressing Beclin-1 siRNAs, but not scrambled oligonucleotides, blocked the effect of rapamycin (fEPSPs at 50–60 min after LFS normalized to the pre-stimulation baseline: 102.5 ± 4.2% in scramble slices treated with rapamycin, 48.0 ± 5.2% in scramble slices treated with vehicle, two-tailed Student’s *t* test, *p* = 0.000001; 48.5 ± 4.4% in Beclin-1 siRNA slices treated with rapamycin, 46.4 ± 4.7% in Beclin-1 siRNA slices treated with vehicle, two-tailed Student's *t* test, *p* = 0.75; Fig. 2e, f). Hence, rapamycin inhibits LTD via autophagy.

To corroborate the necessity of autophagy inhibition in LTD, we treated slices with trehalose, an mTOR-independent autophagy inducer^43–45^. Consistent with the reports that trehalose activates autophagy and also blocks autophagic flux^46^, trehalose increased both LC3-II and p62 (Supplementary Fig. 1A–C). Trehalose blocked LTD but not LTP (fEPSPs at 70–80 min after LFS normalized to the pre-stimulation baseline: 102.9 ± 2.0% in trehalose-treated slices, 47.2 ± 0.7% in vehicle-treated slices, two-tailed Student's *t* test, *p* = 3.9 × 10^−15^; fEPSPs at 50–60 min after HFS normalized to the pre-stimulation baseline: 148.8 ± 3.5% in trehalose-treated slices, 152.7 ± 0.9% in vehicle-treated slices, two-tailed Student's *t* test, *p* = 0.27; Fig. 2g, h).

**Fig. 3:**
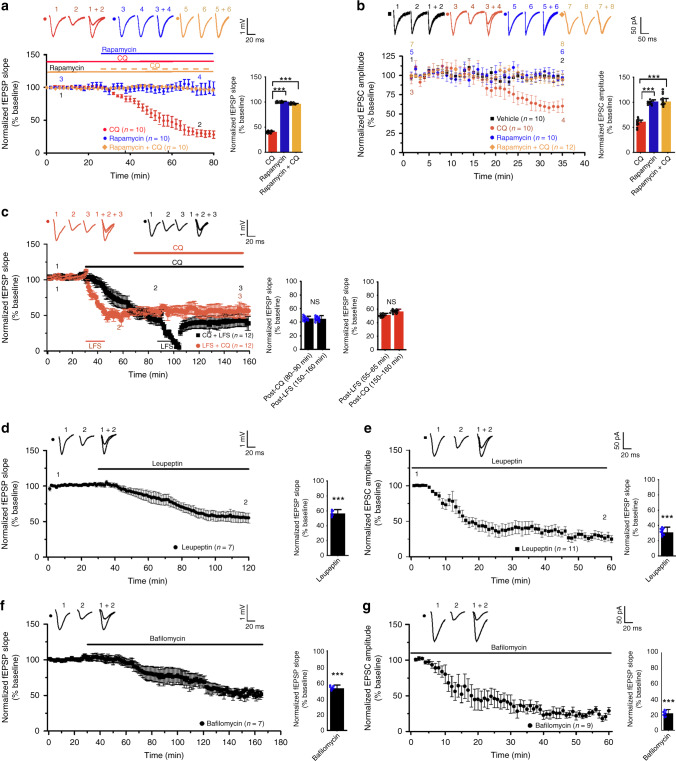
Autophagy inhibitors induce synaptic depression. **a** Field excitatory postsynaptic potentials (fEPSPs) evoked by stimulation at the Schaffer collateral pathway were recorded in the CA1 region of acute hippocampal slices; CQ (120 μM), rapamycin (1 μM), or CQ plus rapamycin was added to the bath; quantification on the right shows the average slope of fEPSPs recorded at 50–60 min after drug perfusion normalized to the pre-treatment baseline for the CQ and the rapamycin group, or to the pre-CQ baseline for the CQ plus rapamycin group; one-way ANOVA was used for statistical analysis and Bonferroni was used for post hoc analysis. **b** Excitatory postsynaptic currents (EPSCs) recorded in CA1 neurons in whole-cell mode; CQ (120 μM), rapamycin (1 μM), or CQ plus rapamycin was infused through the patching pipette; quantification on the right shows the average amplitude of EPSCs at 30–35 min normalized to that recorded at 0–5 min after breaking in; one-way ANOVA was used for comparison across groups; Bonferroni test was used for post hoc analysis. **c** CQ (120 μM) and LFS were applied sequentially to slices; quantification on the right shows the average slope of fEPSPs (recorded in designated time frames after the beginning of recording) normalized to the pre-treatment baseline; two-tailed paired t-test was used for statistical analysis. Slices were perfused with leupeptin (300 μM; **d**) or bafilomycin A1 (20 μM; **f**); quantification on the right shows the average slope of fEPSPs recorded at 80–90 min after drug perfusion normalized to the pre-treatment baseline; two-tailed Student’s *t* test was used for statistical analysis. CA1 neurons were infused with leupeptin (300 μM; **e**) or bafilomycin A1 (20 μM; **g**); quantification on the right shows the average amplitude of EPSCs at 35–40 min normalized to that recorded at 0–5 min after breaking in; two-tailed Student’s *t* test was used for statistical analysis. Data are presented as mean ± SEM; *n* indicates the number of slices (one slice from each animal); ***p < 0.001; no adjustments were made for multiple comparisons. Source data are provided as a Source Data file.

To test whether autophagy inhibition is sufficient for synaptic depression, we inhibited autophagy with chloroquine (CQ). CQ increased LC3-II and p62 in hippocampal slices, consistent with inhibition of autophagic flux (Supplementary Fig. 1A–C). Postsynaptic responses in CA1 neurons were reduced by CQ either added to the bath solution during field potential recording or infused -via the patch pipette during whole-cell recording [fEPSP was reduced by 60.55 ± 3.81% at 50–60 min after adding CQ to the bath, two-tailed paired Student’s *t* test, *p* = 0.0001 for before vs. after CQ treatment; EPSC was reduced by 34.65 ± 0.65% at 30–35 min after breaking the cell membrane, two-tailed paired t-test, *p* = 0.0001 for comparison with the baseline]. The effect of CQ was blocked by rapamycin in field recording [fEPSPs at 50–60 min after CQ treatment normalized to the pre-CQ baseline: 100.5 ± 1.0% for rapamycin, 39.5 ± 2.5% for CQ, 95.3 ± 1.7% for CQ plus rapamycin; one-way ANOVA (*p* = 6.5 × 10^−20^) and Bonferroni for post hoc analysis (*p* = 4.3 × 10^−18^ for CQ plus rapamycin vs. CQ); Fig. 3a] and in whole-cell recording [EPSCs at 30–35 min after breaking in normalized to baseline: 39.5 ± 2.5% for CQ, 100.3 ± 7.3% for CQ plus rapamycin, 100.5 ± 1.0% for rapamycin; one-way ANOVA (*p* = 5.5 × 10^−10^) and Bonferroni for post hoc analysis (*p* = 3.6 × 10^−9^ for CQ plus rapamycin vs. CQ; Fig. 3b]. Hence, autophagy inhibition in postsynaptic neurons is sufficient to induce synaptic depression.

Next, we applied CQ and LFS sequentially to test whether they induce synaptic depression via the same pathway. fEPSPs were reduced by CQ (fEPSP at 50–60 min after CQ treatment: 44.7 ± 4.0%; two-tailed Student’s *t* test was used for comparison with pre-CQ baseline, *p* = 0.0001) and not further decreased by LFS (fEPSP at 50–60 min post LFS: 44.38 ± 5.3%; two-tailed Student's *t* test was used for comparison with 50–60 min after CQ treatment, *p* = 0.96; Fig. 3c). Likewise, CQ treatment following LFS did not further decrease fEPSP (fEPSP at 10–20 min post-LFS: 50.34 ± 3.3%; fEPSP at 80–90 min post-CQ: 55.57 ± 4.5; two-tailed Student's *t* test, *p* = 0.35; Fig. 3c). These results indicate that CQ and LFS use a common pathway to induce synaptic depression.

To validate the effect of autophagy inhibition on synaptic responses, we treated slices with leupeptin and bafilomycin A1 (a vacuolar H^+^-ATPase inhibitor blocking the fusion between autophagosomes and lysosomes). Bafilomycin A1 inhibits autophagic flux in hippocampal slices (Supplementary Fig. 1A–C). Like CQ, bafilomycin A1 and leupeptin reduced synaptic response (fEPSP after leupeptin treatment: 55.8 ± 5.9%, *p* = 0.000001 vs. baseline; EPSC after breaking in to infuse leupeptin: 30.4 ± 7.3%, *p* = 9 × 10^−9^ vs. baseline; fEPSP after bafilomycin A1 treatment: 52.4 ± 4.6%, *p* = 8.3 × 10^−7^ vs. baseline; EPSC after breaking in to infuse bafilomycin A1: 21.4 ± 5.4%, *p* = 1.4 × 10^−10^ vs. baseline; two-tailed Student’s *t* test; Fig. 3d–g).

Taken together, these findings indicate that autophagy inhibition in postsynaptic CA1 neurons is both necessary and sufficient for LTD induction.

### AMPAR internalization in LTD requires autophagy inhibition

**Fig. 4:**
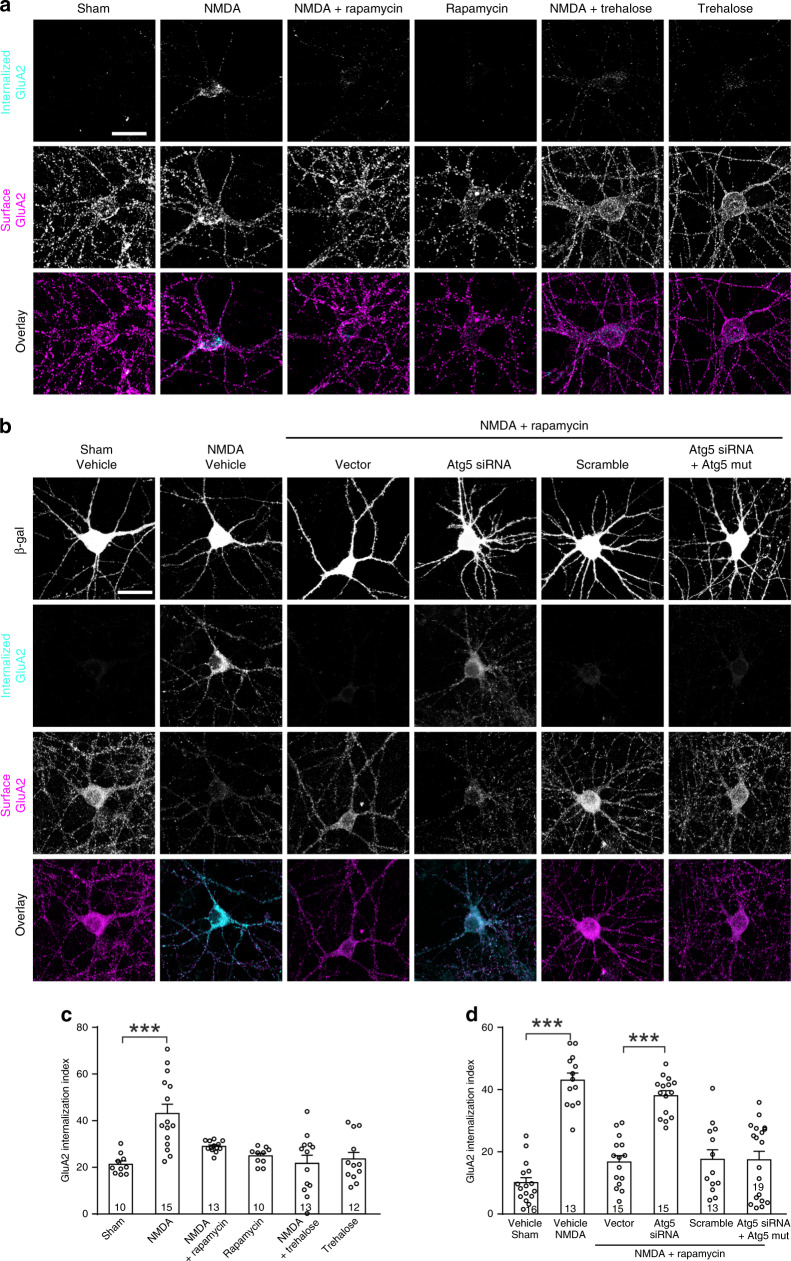
Autophagy inhibition is required for AMPA receptor internalization during LTD. Untransfected primary hippocampal neurons (**a**, **c**) and neurons transfected with designated constructs (**b**, **d**) were treated with NMDA (30 μM, 5 min), rapamycin (1 μM, 10 min) and trehalose (20 mM, 30 min) alone or in combination as indicated. **a**, **b** Representative images; scale bar, 20 μm. **c** Quantification of GluA2 internalization index [(integrated fluorescence intensity of internalized GluA2)/(total integrated fluorescence intensity of internalized and surface GluA2)] in **a**; Kruskal–Wallis one-way ANOVA on ranks was used to compare across groups (*p* = 0.000062); groups significantly different from the sham group were identified with Dunn’s test and marked with asterisks. **d** Quantification of GluA2 internalization in **b**; Kruskal–Wallis one-way ANOVA on ranks was used to compare across groups (*p* = 8.5402 × 10^−11^); Dunn’s test was used to identify significantly different groups; no adjustments were made for multiple comparisons. The graph shows mean ± SEM; the number in the bar indicates the number of cells; ***p < 0.001. Source data are provided as a Source Data file.

LTD expression is mediated by the removal of AMPA receptors from synapses^14,17,47,48^. To test whether autophagy is involved in AMPA receptor internalization, cultured hippocampal neurons (DIV 17) were stimulated with NMDA (30 μM, 5 min) to induce chemical LTD which shares molecular mechanisms with LFS-induced LTD^49^. The internalization of AMPA receptor subunit GluA2 was analyzed by using an antibody-based internalization assay^15,16^. NMDA-induced GluA2 internalization and this was blocked by rapamycin (Fig. 4a, c). Rapamycin treatment alone had no effect on GluA2 internalization and activated autophagy (Fig. 4a, c; Supplementary Fig. 2A–C).

To examine whether rapamycin blocks GluA2 internalization via autophagy, we transfected neurons with plasmids expressing Atg5 siRNAs. Atg5 siRNAs (but not a scrambled sequence) knocked down overexpressed Atg5 (but not Beclin-1) and this effect was abolished by co-expressing Atg5 resistant to Atg5 siRNAs (Atg5mut, carrying synonymous mutations in the siRNA binding site; Supplementary Fig. 2D, E). The Atg5 siRNA also effectively knocked down endogenous Atg5 (Supplementary Fig. 2F, G).

Atg5 siRNAs blocked the effect of rapamycin on NMDA-induced GluA2 internalization, and the blockade was obliterated by Atg5mut (Fig. 4b, d). Basal GluA2 internalization was unchanged by Atg5 siRNAs (Supplementary Fig. 2H, I). Scrambled siRNAs had no effect on NMDA-induced or basal GluA2 internalization (Fig. 4b, d; Supplementary Fig. 2H, I). Hence, rapamycin blocks NMDA-induced GluA2 internalization through autophagy. Moreover, trehalose which increased both LC3-II and p62 as in hippocampal slices blocked NMDA-induced but not basal GluA2 internalization (Supplementary Fig. 2A–C, Fig. 4a, c). These results indicate that autophagy inhibition is required for AMPA receptor internalization during LTD.

To test whether inhibiting autophagy is sufficient to induce AMPA receptor internalization, we treated hippocampal neurons with CQ. CQ increased both LC3-II and p62 in cultured neurons (Supplementary Fig. 2A–C). It increased GluA2 internalization and occluded the effect of NMDA on GluA2 internalization (Supplementary Fig. 3). Hence, CQ and NMDA induce GluA2 internalization via the same pathway. Moreover, we inhibited autophagy with bafilomycin A1 and leupeptin. Both increased LC3-II, p62, and GluA2 internalization in neurons (Supplementary Fig. 2A–C; Supplementary Fig. 3). These results indicate that autophagy inhibition is sufficient to induce GluA2 internalization.

Taken together, these findings indicate that autophagy inhibition is both necessary and sufficient for AMPA receptor internalization in LTD.

### Autophagy regulates endocytic recycling in LTD

**Fig. 5:**
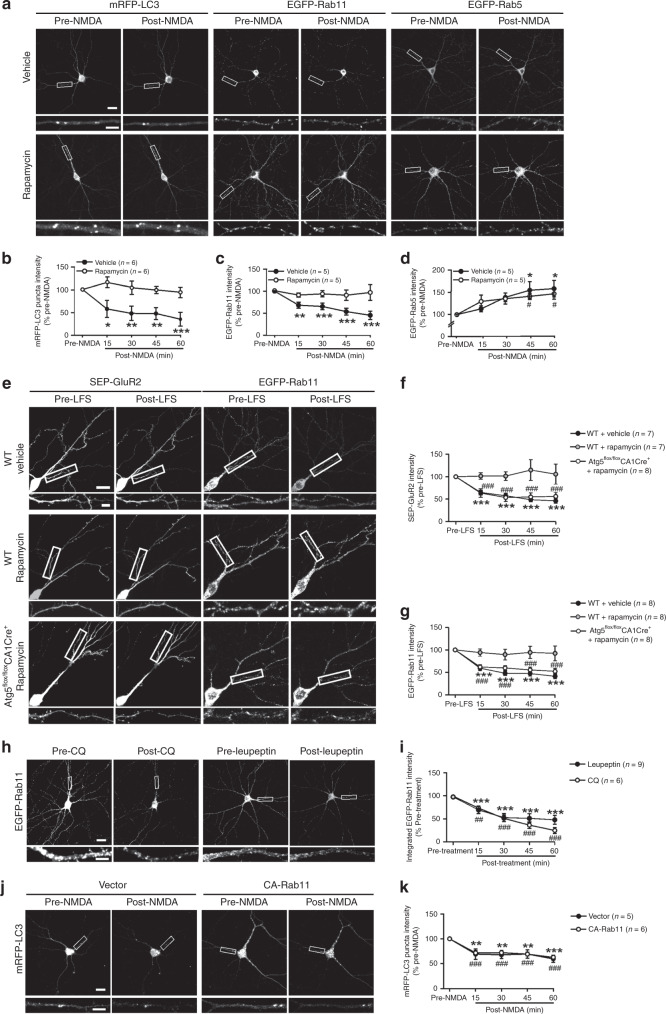
Autophagy inhibition in LTD causes a decrease in recycling endosomes. **a** Representative images of the same neuron before and after NMDA treatment. **b**–**d** Quantification of **a**; one-way RM ANOVA was used to compare across time points (vehicle: *p* = 0.001 in **b**, *p* = 0.000004 in **c**, *p* = 0.014 in **d**; rapamycin: *p* = 0.199 in **b**, *p* = 0.927 in **c**, *p* = 0.006 in **d**); time points significantly different from the pre-stimulation baseline were identified with Holm–Sidak test; *p < 0.05, **p < 0.01, ***p < 0.001 for the vehicle group in **b**–**d**; ^#^*p* < 0.05, ^##^*p* < 0.01 for the rapamycin group in **d**. **e** Representative images of the same neuron in hippocampal slices before and after LFS (low-frequency stimulation). **f, g** Quantification for **e**; one-way RM ANOVA was used to compare across time points (**f**: WT + vehicle, *p* = 4.86 × 10^−12^; WT + rapamycin, *p* = 0.909; *Atg5*^*flox/flox*^*CA1Cre*^*+*^ + rapamycin, *p* = 2 × 10^−6^. **g**: WT + vehicle, *p* = 3.87 × 10^−10^; WT + rapamycin, *p* = 0.900; *Atg5*^*flox/flox*^*CA1Cre*^*+*^ + rapamycin, *p* = 2.68 × 10^−10^). **h, j** Representative images of the same neuron before and after treatment. **i** Quantification of **h**; one-way RM ANOVA was used to compare across time points (*p* = 1.0073 × 10^−11^ for CQ and *p* = 5.85 × 10^−7^ for leupeptin); Holm–Sidak test was used to identify time points significantly different from the pre-stimulation baseline; ***p < 0.001 for CQ; ^###^*p* < 0.001 for leupeptin. **k** Quantification of **j**; one-way RM ANOVA was used to compare across time points (*p* = 0.00018 for vector, *p* = 0.000003 for CA-Rab11); Holm–Sidak test was used to identify time points significantly different from pre-stimulation; ***p < 0.001 for vector-transfected cells; ^###^*p* < 0.001 for CA-Rab11 transfected cells. Data are presented as mean ± SEM; *n* indicates the number of cells in **b**–**d** and **h**–**k**, and the number of slices in **f** and **g**; no adjustments were made for multiple comparisons. Scale bar: 20 μm in top and 5 μm in lower images. Source data are provided as a Source Data file.

Since AMPA receptors are transported in endosomes during LTD, we proceeded to test whether autophagy affects endosomal trafficking. We transfected hippocampal neurons (DIV 14) with plasmids expressing EGFP-tagged Rab proteins to label endosomes (Rab5 for early endosomes, Rab11 for recycling endosomes, Rab9 for late endosomes) along with mRFP-LC3. Neurons were imaged 3–5 days later before and after chemical LTD induction. NMDA decreased mRFP-LC3 puncta and Rab11-labeled recycling endosomes, increased Rab5-labeled early endosomes, and had no significant effect on Rab9-labeled late endosomes (Fig. 5a–d; Supplementary Fig. 4A, B).

To test whether autophagy inhibition contributes to the endosomal changes, we co-treated neurons with NMDA and rapamycin. While rapamycin had no effect on basal Rab11-labeled recycling endosomes (Supplementary Fig. 4C, D), it blocked their changes and mRFP-LC3 puncta decrease induced by NMDA (Fig. 5a–c). Rapamycin had no significant effect on Rab5-labeled early endosomes in untreated or NMDA-stimulated cells (Supplementary Fig. 4C, D; Fig. 5a, d). Trehalose and NMDA co-treatment had the same effect on endosomes as rapamycin and NMDA (Supplementary Fig. 4E, F). These results indicate that autophagy inhibition in LTD is required to decrease endocytic recycling.

To consolidate this finding, we examined endosomes using live imaging in cultured hippocampal slices. In slices transfected with a construct expressing GluA2 tagged with Super Ecliptic pHluorin (SEP, displaying fluorescence only when it is on the cell surface)^50,51^, LFS decreased GluA2 fluorescence, suggestive of GluA2 internalization (Fig. 5e, f). This decrease was blocked by rapamycin (Fig. 5e, f). LFS decreased Rab11 labeled recycling endosomes, increased Rab5 labeled early endosomes, and had no significant effect on Rab9 labeled late endosomes (Fig. 5e, g; Supplementary Fig. 4G–J). Rapamycin blocked LFS-induced alteration of Rab11 but not that of Rab5 (Fig. 5e, g; Supplementary Fig. 4G, H). In Atg5 knockout slices, rapamycin had no effect on LFS-induced GluA2 internalization and the recycling endosome decrease (Fig. 5e–g), indicating that LFS induces AMPA receptor internalization and reduces recycling endosomes in an autophagy-dependent manner. Moreover, CQ and leupeptin decreased Rab11-labeled recycling endosomes (Fig. 5h, i), indicating that autophagy inhibition is sufficient to decrease recycling endosomes. To test whether the reduction of recycling endosomes contributes to autophagy inhibition, we used constitutively active Rab11 (CA-Rab11) to abate the recycling endosome decrease. CA-Rab11 increased transferrin uptake (Supplementary Fig. 5A, B), confirming that it promotes endocytic recycling. Despite more recycling endosomes, mRFP-LC3 puncta still decreased (Fig. 5j, k). Hence, autophagy inhibition in LTD is not caused by the reduction of recycling endosomes.

Fewer recycling endosomes in NMDA-treated cells suggest reduced endosomal recycling. This is confirmed by the transferrin assay (Supplementary Fig. 5C, D). NMDA-induced decrease in transferrin uptake was blocked by rapamycin, and the rapamycin effect was obliterated by Atg5 siRNAs (Supplementary Fig. 5C, D). Hence, endosomal recycling is reduced by NMDA treatment through autophagy inhibition.

**Fig. 6:**
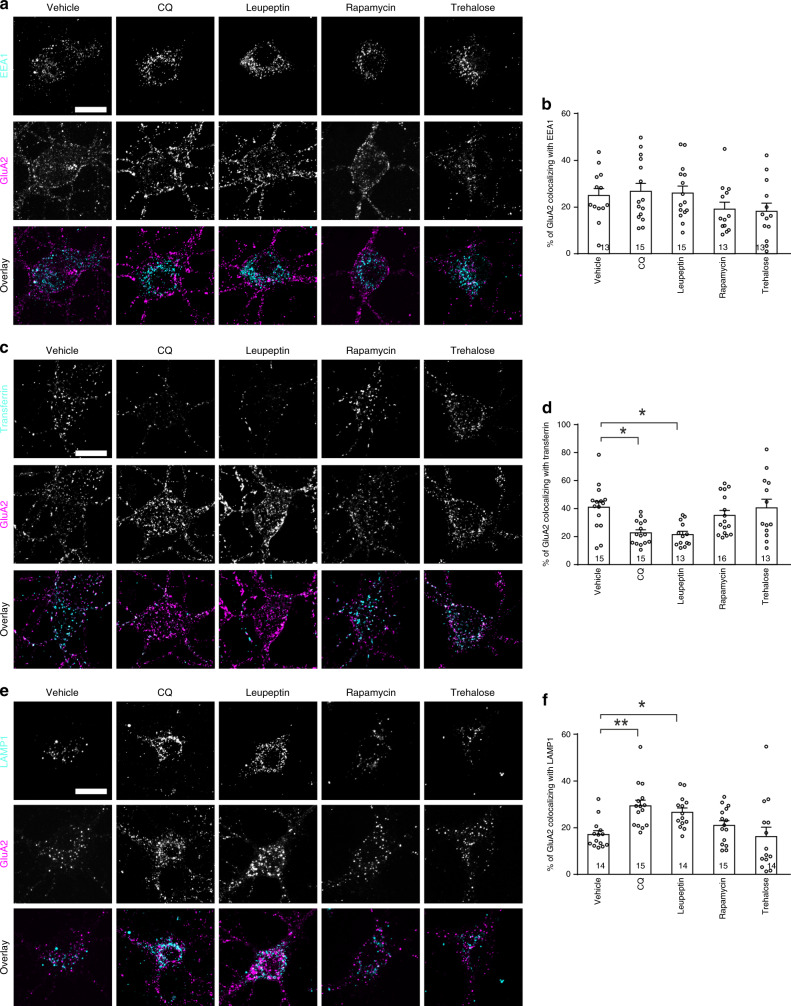
The effect of autophagy activators and inhibitors on endocytic trafficking of GluA2. **a**, **c**, **e** Representative images of primary hippocampal neurons incubated with the GluA2 antibody alone or along with transferrin for 15 min, and then fixed for immunostaining; CQ (120 μM, 10 min), leupeptin (300 μM, 30 min), rapamycin (1 μM, 10 min), or trehalose (20 mM, 30 min) were added to the medium during antibody incubation. **b** Quantitation for the percentage of GluA2 colocalized with EEA1 in **a**; Kruskal–Wallis one-way ANOVA on ranks was used to compare across groups (*p* = 0.131). **d** Quantitation for the percentage of GluA2 colocalized with transferrin in **c**; Kruskal–Wallis one-way ANOVA on ranks was used to compare across groups (*p* = 0.002), and Dunn’s test was used for post hoc analysis. **f** Quantitation for the percentage of GluA2 colocalized with LAMP1 in **e**; Kruskal–Wallis one-way ANOVA on ranks was used to compare across groups (*p* = 0.000534), and Dunn’s test was used for post hoc analysis. Data are presented as mean ± SEM; *p < 0.05, **p < 0.01; no adjustments were made for multiple comparisons; *n* in the bar indicates the number of cells in each group. Scale bar, 20 μm. Source data are provided as a Source Data file.

To test if autophagy affects the endocytic trafficking of GluA2, we assessed the colocalization of GluA2 with EEA1 (an early endosome marker), LAMP1 (a late endosome marker), or transferrin. Hippocampal neurons (DIV 17) were incubated with the GluA2 antibody alone or along with transferrin. After rinse to remove unbound antibodies and transferrin, neurons were left 15 min to allow for transferrin and antibody-labeled surface GluA2 to be internalized. GluA2 was detected in EEA1, transferrin, and lamp1 positive structures (Fig. 6). Rapamycin and trehalose had no significant effect on the distribution of GluA2 in endosomes (Fig. 6). CQ and leupeptin decreased GluA2 in recycling endosomes, increased GluA2 in late endosomes, and had no effect on GluA2 in early endosomes (Fig. 6).

We also examined GluA2 distribution in Atg5^flox/flox^ neurons transduced with AAV Cre which had decreased Atg5 expression (Supplementary Fig. 6A, B). These neurons had less GluA2 in recycling endosomes, more GluA2 in late endosomes, and no change to GluA2 in early endosomes (Supplementary Fig. 6C–F). These results indicate that autophagy inhibition reduces GluA2 in recycling endosomes.

To test if Rab11-containing recycling endosomes are involved in the endocytic trafficking of GluA2, we transfected cultured neurons (DIV 14) with the EGFP-Rab11 construct and analyzed GluA2 in endocytic trafficking 3 days later. A fraction of internalized GluA2 colocalized with Rab11, and the colocalization was increased by NMDA (Supplementary Fig. 6G, H). Hence, GluA2 is transported to Rab11-containing recycling endosomes during LTD.

**Fig. 7:**
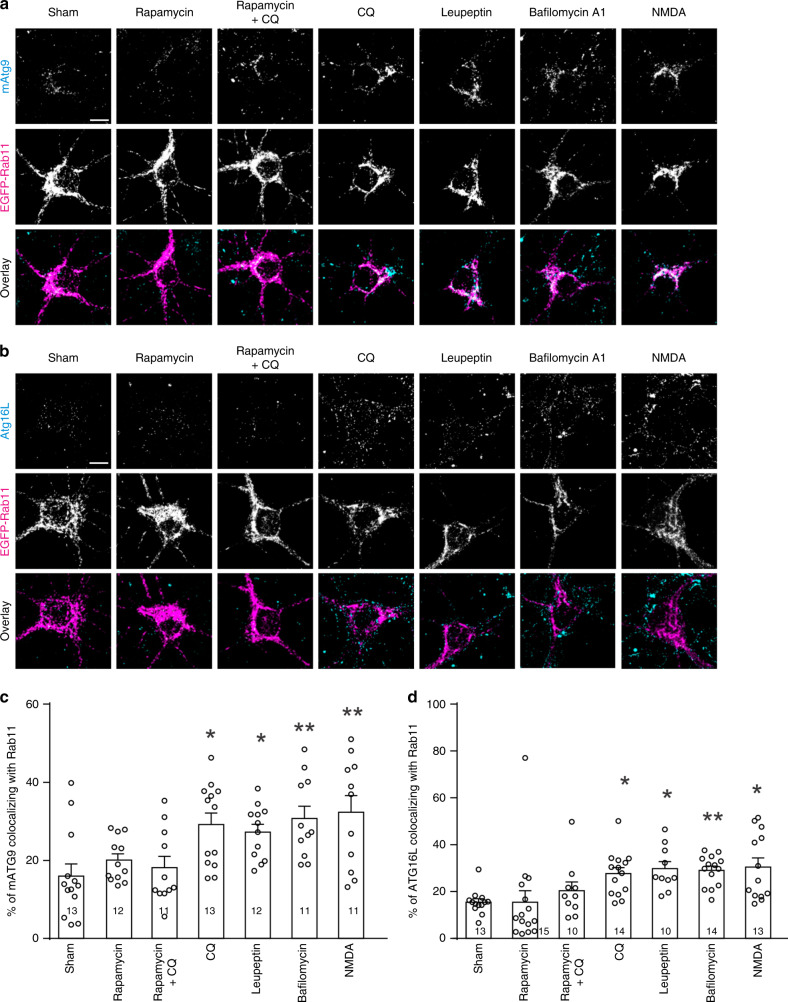
Autophagy inhibition leads to an increase in Atg16L and Atg9 in recycling endosomes. **a**, **b** Representative images of primary hippocampal neurons treated with rapamycin (1 μM, 10 min), leupeptin (300 μM, 30 min), bafilomycin A1 (20 μM, 30 min), NMDA (30 μM, 5 min), CQ (120 μM, 10 min), or CQ along with rapamycin (1 μM, pretreated for 20 min); scale bar, 10 μm. **c** Quantitation of the percentage of mAtg9 colocalized with Rab11 in **a**; one-way ANOVA was used to compare across groups (*p* = 0.000186), and Student–Newman–Keuls test was used to identify groups significantly different from the sham group. **d** Quantitation of the percentage of Atg16L colocalized with Rab11 in **c**; Kruskal–Wallis one-way ANOVA on ranks was used to compare across groups (*p* = 5 × 10^−6^), and Dunn’s test was used to identify groups significantly different from the sham group. Data are presented as mean ± SEM; *p < 0.05, **p < 0.01; no adjustments were made for multiple comparisons; *n* in the bar indicates the number of cells in each group. Source data are provided as a Source Data file.

Why endocytic recycling is reduced when autophagy is inhibited? Atg16L and mAtg9A-containing endocytic vesicles resulting from endocytosis from plasma membranes meet in recycling endosomes to form autophagosome precursors^52,53^. Since Atg16L and mAtg9 in recycling endosomes are en route to autophagosomes, inhibition of autophagic flux may cause accumulation of Atg16L and mAtg9 in recycling endosomes, thereby reducing endocytic recycling. Indeed, the proportion of Atg16L and mAtg9 in Rab11 positive structures increased in cells treated with CQ, leupeptin or bafilomycin A1, and it was unchanged by rapamycin or co-treatment with rapamycin and CQ (Fig. 7). NMDA also increased Atg16L and mAtg9 in Rab11 labeled recycling endosomes (Fig. 7).

Taken together, these findings indicate that autophagy inhibition during LTD leads to a suppression of endosomal recycling, which presumably facilitates the removal of AMPA receptors from plasma membranes.

### Autophagy is inhibited by active caspase-3 in LTD

**Fig. 8:**
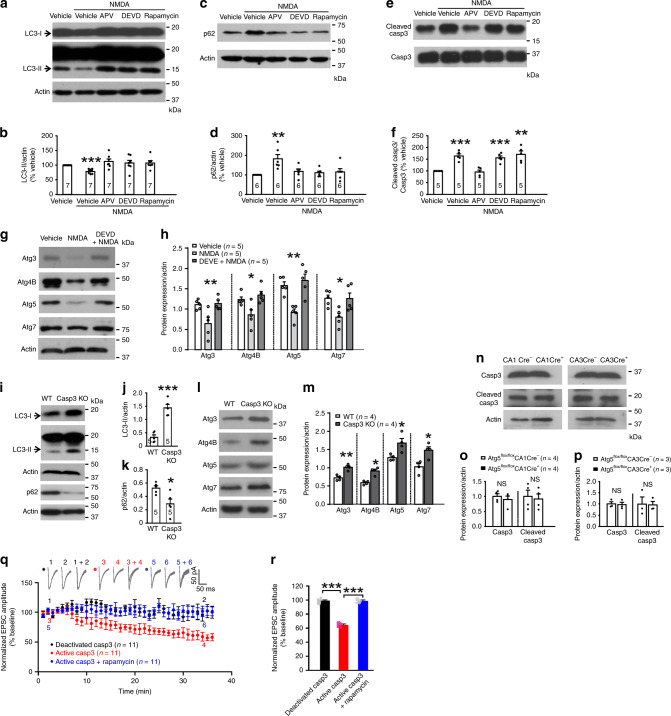
Autophagy inhibition in LTD requires caspase-3 activity. **a**, **c**, **e** Representative blots of primary hippocampal neurons lysed 15 min after NMDA treatment; DEVD (5 μM), APV (100 μM), rapamycin (1 μM), 5 min pretreatment. **b** Quantification for **a**. **d** Quantification for **c**. **f** Quantification for **e**. **b**, **d**, **f** Kruskal–Wallis one-way ANOVA on ranks was used for statistical analysis (*p* = 0.009 in **b**, *p* = 0.009 in **d**, *p* = 0.001 in **f**), and Student–Neuman–Keuls test for comparison with vehicle. **g** Representative blot of primary hippocampal neurons lysed 30 min after NMDA treatment. **h** Quantification for **g**; one-way ANOVA was used for statistical analysis (*p* = 0.007 for Atg3, *p* = 0.012 for Atg4B, *p* = 0.000847 for Atg5, *p* = 0.017 for Atg7), and Student–Neuman–Keuls test for comparison with vehicle. **i**, **l** Representative immunoblots of the CA1 region (P17–19). **j** Quantification of LC3-II in **i** (*p* = 0.000186). **k** Quantification of p62 in **i** (*p* = 0.0327). **m** Quantification for **l** (*p* = 0.00889 for Atg3, *p* = 0.0203 for Atg4B, *p* = 0.0323 for Atg5, *p* = 0.0275 for Atg7); two-tailed pared t-test was used for statistical analysis in **j**, **k**, and **m**. **n** Representative blot of the CA1 region. **o** Quantification for the CA1 lysate in **n**. **p** Quantification for the CA3 lysate in **n**. Two-tailed paired t-test or Wilcoxon Signed Rank Test was used for full-length caspase-3 (*p* = 0.479 in **o**, *p* = 0.867 in **p**) and cleaved caspase-3 (*p* = 0.375 in **o**, *p* = 0.806 in **p**). **q**, **r** Whole-cell recordings of CA1 neurons; caspase-3 (2 ng/μl), rapamycin (10 μM), deactivated caspase-3 (2 ng/μl). (R) EPSCs at 30–35 min were normalized to the 0–5 min baseline; one-way ANOVA was used to compare across groups (*p* = 3.6 × 10^−18^), and Bonferroni test for post hoc analysis. The number in the bars and *n* represent the number of cell cultures (**b, d**, **f**, **h**), animals (**j**, **k**, **m**, **o**, **p**), or cells (**q**); no adjustments for multiple comparisons. Data show mean ± SEM. *p < 0.05, **p < 0.01, ***p < 0.001. Source data are provided as a Source Data file.

NMDA receptor-dependent LTD and AMPA receptor internalization in hippocampal neurons require caspase-3 activation^16^. To examine the relationship between autophagy and caspase-3 in LTD, we treated cultured neurons with NMDA along with rapamycin or the caspase-3 inhibitor DEVD-fmk. The effects of NMDA on LC3-II and p62 were blocked by DEVD-fmk (Fig. 8a–d). Rapamycin, by contrast, had no effect on NMDA-induced increase in cleaved, active caspase-3 (Fig. 8e, f). Both caspase-3 activation and autophagy inhibition induced by NMDA were abolished by the NMDA receptor antagonist (2R)-amino-5-phosphonopentanoate (AP5; Fig. 8a–f). These results indicate that autophagy inhibition in LTD requires caspase-3 activity.

Several Atg proteins are caspase-3 substrates^54^. In hippocampal slices treated with NMDA, the reported caspase-3 substrates Atg3, 4B, 5, and 7 decreased, and the decrease was blocked by DEVD-fmk (Fig. 8g, h), suggesting that caspase-3 activation in LTD causes proteolysis of Atg proteins. To confirm that caspase-3 indeed inhibits autophagy, we measured LC3-II and p62 in caspase-3 knockout mice. LC3-II increased, while p62 decreased in the CA1 region of caspase-3 knockout mice (Fig. 8i–k). Also, Atg3, 4B, 5, and 7 increased in caspase-3 knockout mice (Fig. 8l, m). In the CA1 region of Atg5^flox/flox^CA1Cre^+^ mice and the CA3 region of Atg5^flox/flox^CA3Cre^+^ mice, full-length and cleaved caspase-3 were unchanged (Fig. 8n–p). These results suggest that caspase-3 inhibits autophagy, at least in part, by cleaving Atg proteins.

To test whether autophagy mediates the function of caspase-3 in LTD, we infused active caspase-3 alone or along with rapamycin via the whole-cell patch pipette into CA1 neurons. Active caspase-3 decreased EPSCs and this effect was obliterated by co-infusion with rapamycin [average EPSCs at 30–35 min normalized to that at 0–5 min after break-in: 64.59 ± 1.44% for active caspase-3 and 98.42 ± 1.56% for active caspase-3 plus rapamycin, one-way ANOVA (*p* = 3.6 × 10^−18^) and post hoc Bonferroni (*p* = 1 × 10^−16^ for active caspase-3 vs. active caspase-3 plus rapamycin); Fig. 8q, r]. Heated, deactivated caspase-3 or rapamycin alone had no effect on EPSCs [98.82 ± 1.10% for deactivated caspase-3 and 99.99 ± 0.59% for rapamycin, post hoc Bonferroni (*p* = 7.4 × 10^−17^ for deactivated caspase-3 vs. active caspase-3); Fig. 8q, r; Fig. 3b]. Hence, autophagy inhibition is required for caspase-3-induced synaptic depression.

To test if caspase-3 activity is required for the recycling endosome decrease in LTD, we transfected cultured hippocampal neurons with the Rab11 construct and induced chemical LTD in the presence of DEVD-fmk. Rab11-labeled recycling endosomes were unchanged by NMDA (Supplementary Fig. 7), indicating that caspase-3 activity is required to reduce endocytic recycling in LTD.

Taken together, these findings indicate that autophagy is inhibited by caspase-3 and mediates the function of caspase-3 in LTD.

### Autophagy increases in adults to reduce LTD inducibility

**Fig. 9:**
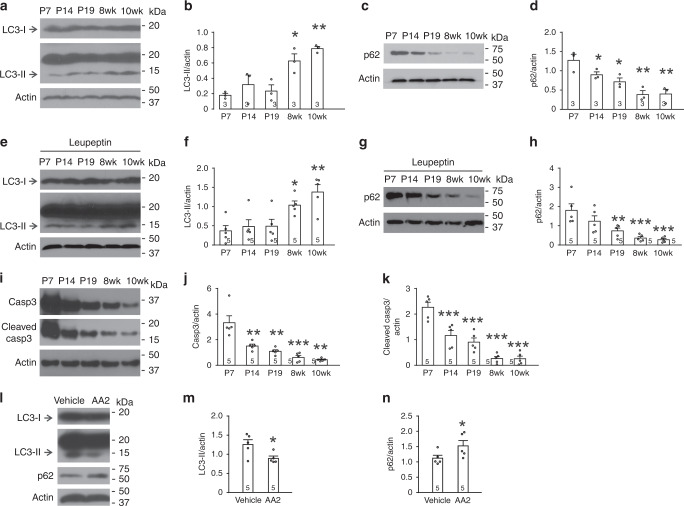
Autophagy increases and caspase-3 decreases in the adult CA1 region. **a**, **c**, **e**, **g**, **i**, **l** Representative blots of the CA1 region from acute hippocampal slices. **b** Quantification for **a**; one-way ANOVA was used to compare across ages (*p* = 0.001), and Student–Newman–Keuls test was used to identify ages significantly different from postnatal day 7 (P7). **d** Quantification for **c**; one-way ANOVA was used to compare across ages (*p* = 0.001), and Student–Newman–Keuls test was used to identify ages significantly different from P7. **f** Quantification for **e**; one-way ANOVA was used to compare across ages (*p* = 0.000903), and Student–Newman–Keuls test was used to identify ages significantly different from P7. **h** Quantification for **g**; one-way ANOVA was used to compare across groups (*p* = 0.000229), and Student–Newman–Keuls test was used to identify ages significantly different from P7. **j**, **k** Quantification for **i**; Kruskal–Wallis one-way ANOVA on ranks or one-way ANOVA was used to compare across groups (*p* = 0.000347 for **j**, *p* = 7.6141 × 10^−8^ for **k**), and Student–Newman–Keuls test was used to identify ages significantly different from P7. **l** Representative blots of the CA1 region of adult wild-type mice (10-weeks of age) treated with AA2 (20 µM) or vehicle. (**m**, **n**) Quantification for **l**; two-tailed paired t-test was used for statistical analysis (*p* = 0.0292 for **m**, *p* = 0.0202 for **n**). The number in the bars and *n* represent the number of animals; no adjustments were made for multiple comparisons. Data show mean ± SEM. *p < 0.05, **p < 0.01, ***p < 0.001. Source data are provided as a Source Data file.

The inducibility of NMDAR-LTD in SC synapses is much lower in adults than in juveniles^10,12,13^. To test if autophagy is involved in this phenomenon, we first examined the developmental pattern of autophagy in the CA1 region. LC3-II increased, while p62 decreased in adulthood (Fig. 9a–d). This developmental change was also present in hippocampal slices treated with leupeptin (Fig. 9e–h). By contrast, both full-length and active caspase-3 decreased in adulthood (Fig. 9i–k). To test whether the caspase decrease in adult hippocampi contributes to the autophagy increase, we treated adult hippocampal slices (10-week-old) with the cell-permeable caspase-3 activator AA2. AA2 decreased LC3-II and increased p62 (Fig. 9l–n). Hence, the autophagy increase in adults is attributable, at least in part, to diminished caspase-3.

**Fig. 10:**
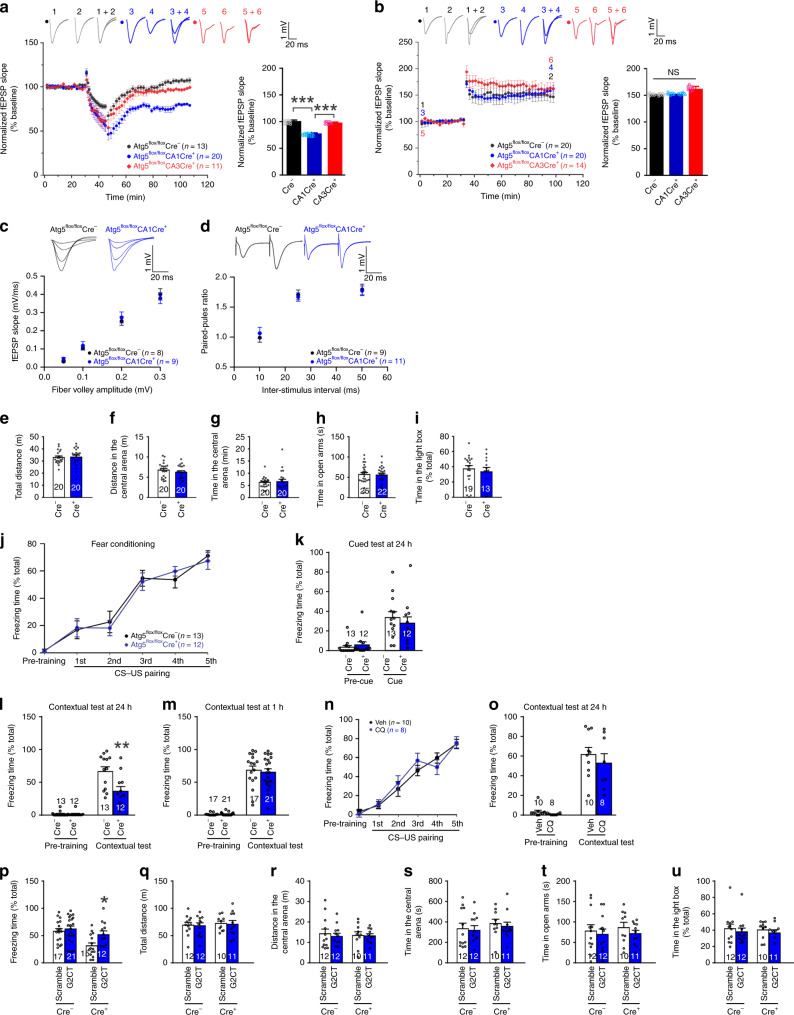
Contextual fear memory is impaired in CA1-specific Atg5 knockout mice. LTD induced by LFS (**a**) and LTP induced by HFS (**b**) in the hippocampal CA1 region of 8-week-old mice; quantification on the right shows fEPSPs at 50–60 min after LFS or HFS normalized to the pre-stimulation baseline; one-way ANOVA was used for comparison across groups (*p* = 3.8 × 10^−28^) and Student–Newman–Keuls test for comparison with the Cre^−^ group. **c** The input-output relationship. **d** Paired-pulse ratio. **e**–**g** Open field test. **h** Elevated zero maze test. **i** Light/dark box test. **j** The percentage of time spent in freezing before and after each CS-US pairing. **k** Cued fear memory tested at 24 h after fear conditioning. **l** Contextual fear memory tested at 24 h after fear conditioning; two-way ANOVA was used for statistical analysis (*p* = 0.005 for interaction between the effects of genotype and test on freezing; *p* = 0.004 for the simple main effect of Cre^−^ vs. Cre^+^ in the contextual test). **m** Contextual fear memory tested at 1 h after fear conditioning. **n, o** Fear conditioning training and contextual memory test at 2 weeks after cannula implantation in the dorsal hippocampal CA1 region of WT mice. CQ was infused through the implanted cannula at 30 min before the contextual fear memory test. **p**–**u** Mice were injected with AAV expressing G2CT or scrambled peptides and tested at 3 weeks after injection for contextual fear memory (**p**; two-way ANOVA was used for statistical analysis; *p* = 0.158 for interaction between the effects of genotype and virus on freezing; *p* = 0.032 for the main effect of virus; *p* = 0.021 for the comparison between scramble vs. G2CT within Cre^+^ using Student–Newman–Keuls test), open field test (**q**–**s**), elevated zero maze test (**t**) or light/dark box test (**u**). Data show mean ± SEM; *p < 0.05, **p < 0.01, ***p < 0.001; no adjustments were made for multiple comparisons; n indicates the number of slices in **a**–**d** (one slice from each animal) and the number of animals in **e**–**u**. Source data are provided as a Source Data file.

We next tested LTD in adult CA1 and CA3-specific Atg5 knockout mice (8 weeks of age). While LFS did not induce noticeable LTD in wild-type or CA3-specific Atg5 knockout slices, it induced LTD in CA1-specific Atg5 knockout slices (fEPSPs at 50–60 min post-LFS normalized to the pre-stimulation baseline: 102.19 ± 0.85% in wild-type slices, 96.1 ± 1.17% in CA3-specific Atg5 knockout slices, 76.82 ± 0.50% in CA1-specific Atg5 knockout slices; one-way ANOVA, *p* = 3.8 × 10^−28^; Fig. 10a). HFS-induced LTP was unchanged in CA1 or CA3-specific Atg5 knockout mice (fEPSPs at 50–60 min post-HFS normalized to the pre-stimulation baseline: 151.4 ± 0.55% in wild-type slices, 151.8 ± 0.91% in CA3-specific Atg5 knockout slices, 151.6 ± 0.81% in CA1-specific Atg5 knockout slices; one-way ANOVA, *p* = 0.08; Fig. 10b). Hence, the inducibility of LTD in adults is restored by Atg5 knockout in postsynaptic neurons. The input-output relationship and paired-pulse ratio were comparable in wild-type and CA1-specific Atg5 knockout slices (Fig. 10c, d).

Taken together, these findings indicate that autophagy in the CA1 region is increased in adulthood and that this increase contributes, at least in part, to a low LTD inducibility.

### Low LTD inducibility in adults is essential for fear memory

Since LTD is required for learning and memory, we examined the behavior of adult CA1-specific Atg5 knockout mice in which LTD can be easily induced (Fig. 10a). Locomotion (the open field test), anxiety-like behavior (the light/dark box and the elevated zero maze test) were comparable in littermates of Cre^+^ and Cre^−^ mice (Fig. 10e–i). During fear conditioning, Cre^+^ and Cre^−^ mice increased freezing behavior with indistinguishable rates (Fig. 10j). At 24 h after fear conditioning, although Cre^+^ and Cre^−^ mice had comparable freezing in the cued fear memory test, Cre^+^ mice froze less in the contextual fear memory test (Fig. 10k, l). The specific impairment of contextual but not cued fear memory is consistent with previous reports that contextual, but not cued fear memory, requires the hippocampus^55^.

To test whether memory acquisition is affected in the knockout mice, we measured contextual fear memory at 1 h after fear conditioning. Knockout and wild-type mice were comparable (Fig. 10m). To test for memory retrieval, we injected the CA1 region with CQ at 30 min before the contextual memory test. CQ and vehicle-injected mice had comparable freezing time during the contextual memory test and fear conditioning (Fig. 10n, o). These findings suggest that the knockout mice have deficits in memory consolidation, but not in learning, memory acquisition, or memory retrieval.

To determine the role of increased LTD inducibility in the contextual fear memory impairment of knockout mice, we generated adeno-associated virus (AAV) expressing G2CT, a peptide inhibiting NMDA receptor-dependent GluA2 internalization and LTD^56,57^. The CA1 region of wild-type littermates of CA1-specific Atg5 knockout mice (8 weeks of age) was injected with 1 μl virus. Four-week G2CT expression caused less freezing in the contextual fear memory test without changing freezing during fear conditioning or cued fear memory test (Supplementary Fig. 8A–C), indicating that G2CT impairs contextual fear memory. LTD was blocked in mice injected with 1 μl AAV G2CT (Supplementary Fig. 8D, E). This finding is consistent with previous reports that LTD is required for contextual fear memory^5,8^.

To allow for LTD expression, we injected less virus and shortened viral expression time. Our tests showed that injection of 200 nl virus and 3-week expression decreased but did not abolish LTD and had no significant effect on contextual fear memory (Supplementary Fig. 8D; Fig. 10l). The injection of 200 nl scramble virus had no effect on LTD (Supplementary Fig. 8E). 200 nl virus transduced fewer cells than 1 µl virus and restored contextual fear memory in Cre^+^ mice without affecting locomotion or anxiety-like behaviors (Fig. 10p–u; Supplementary Fig. 8F). Hence, excessive LTD in CA1-specific Atg5 knockout mice impairs contextual fear memory.

Taken together, these findings indicate that the low inducibility of LTD in adult CA1 neurons is required for the consolidation of contextual fear memory.

## Discussion

Although autophagy has been implicated in neural development and neurotransmitter release, its role in synaptic plasticity is unclear. In this study, we found an unexpected decrease in autophagic flux by NMDA treatment and LFS. This decrease is transient and begins within 15 min after stimulation. An earlier study reports a delayed increase in autophagy that peaks at 2 h after NMDA stimulation while no significant changes at earlier times^29^. Different cell culture and stimulation methods may account for the lack of early autophagy changes in this study. The delayed activation of autophagy, however, is not likely to act on the induction or expression of LTD because it occurs long after these processes have started. This notion is consistent with the findings that autophagy inhibitors have no effect on LFS-induced LTD^30^.

Our results indicate that the early autophagy decrease is both necessary and sufficient for LTD induction. First, we found that rapamycin blocks NMDA receptor-dependent LTD and AMPA receptor internalization. Using Atg5 knockout mice and Atg5 siRNAs, we confirm that rapamycin requires autophagy to take effect. Rapamycin has been shown to inhibit long-lasting LTP (L-LTP) and metabotropic glutamate receptor-dependent LTD (mGluR-LTD) by regulating protein synthesis^29,58–61^. Our study indicates that rapamycin can also influence synaptic plasticity through autophagy. Second, we show that autophagy inhibition induces synaptic depression and AMPA receptor internalization.

In chemical LTD, while rapamycin has no effect on caspase-3 activation, a caspase-3 inhibitor blocks the decrease in autophagic flux, indicating that autophagy is inhibited by caspase-3, presumably through proteolysis. Autophagy inhibition is crucial for the control of postsynaptic responses by caspase-3, as rapamycin obliterates synaptic depression induced by active caspase-3.

What is the specific function of autophagy in NMDAR-LTD? Our study shows that autophagy modifies endocytic recycling. Endocytic recycling is reduced by NMDA receptor activation. This decrease is blocked by rapamycin. Conversely, CQ inhibits endocytic recycling. Consistent with the effect of autophagy on endocytic recycling, NMDA receptor-dependent GluA2 internalization requires autophagy inhibition and CQ promotes GluA2 internalization. These findings suggest that autophagy contributes to AMPA receptor internalization in NMDAR-LTD by modulating endocytic recycling.

Our study indicates that autophagy regulates recycling endosomes during NMDAR-LTD. This is supported by the finding that during LTD, while rapamycin obliterates the recycling endosome decrease, constitutively active Rab11 does not alter the autophagosome decrease. Additionally, autophagy inhibition is sufficient to reduce recycling endosomes. Hence, it is the reduction of autophagy that attenuates endocytic recycling, but not vice versa. This finding does not contradict the known regulation of autophagy biogenesis by recycling endosomes during such conditions as starvation^53^.

We find that autophagy increases with age and that this increase contributes to reduced LTD inducibility in adulthood. These findings suggest that autophagy serves as a mechanism to dampen LTD in adult brains. It is noted that aging switches the role of autophagy in LTD from an inhibitor to a supporter, as autophagy inhibitors reduce LFS-induced LTD in aged brains^30^. The possible reasons for the switch are that LFS induces tau oligomerization in aged but not in adult, non-aged brains, and autophagy is involved in the degradation of tau oligomers^30^.

NMDAR-LTD is required for various hippocampus-dependent contextual fear memory^5,8^. Contextual fear memory is impaired in adult, CA1-specific Atg5 knockout mice. This is due to increased inducibility of NMDAR-LTD because it can be ameliorated by attenuating NMDAR-LTD with the G2CT peptide. These findings indicate that NMDAR-LTD needs to be fine-tuned to optimize memory.

In sum, this study uncovers unrecognized functions of autophagy in synaptic plasticity, endocytic recycling, the age-dependence of LTD induction, and memory consolidation.

## Methods

### Animals, DNA constructs, and reagents

The CA3-specific Cre (*G32-4*) and CA1-specific Cre (*T29-1*) mice were purchased from the Jackson Laboratory. The *Atg5*^*flox*^ mouse was purchased from RIKEN BioResource Center. All animal procedures followed the US National Institutes of Health Guidelines Using Animals in Intramural Research and were approved by the National Institute of Mental Health Animal Care and Use Committee. The EGFP-Rab5 and EGFP-Rab9 constructs were generously provided by Dr. Juan Bonifacino (National Institute of Child Health and Human Development, National Institutes of Health). The following constructs were purchased from Addgene: EGFP-Rab11a-7 (#56444), PmRFP-LC3B (#21075), CaMKIIa iChioC 2A tDimer (#66709), pMXs-puro GFP-p62 (#38277). The constitutively active Rab11 construct was generated by mutating EGFP-Rab11a-7 [Q(CAA)70L(CTG)]. The Atg5 siRNA (GGCTCACTTTATGTCATGT) and scrambled oligonucleotide (GACGTGAACGGATAACACT) were inserted into the BglII/HindIII site of the pSuper plasmid. cDNAs of HA-Atg5, siRNA-resistant Atg5 (G366C, C372T, T375C, G381C) were inserted into the XmaI/KpnI site of the pGW1 vector, HA-Beclin-1 cDNAs were inserted into the BglII/EcoR1 site of the pGW1 vector. The sequences encoding the G2CT (KRMKLNINPS, AAG CGG ATG AAG CTG AAC ATC AAC CCT AGC) and the scrambled peptide (RKNNSKMLIP, CGG AAG AAC AGC AAG ATG CTG ATC CCT) were inserted into the EcoR1/Kpnl site of the CaMKIIa iChioC 2A tDimer plasmid. The mRFP-LC3BG120A plasmid was generated by using Phusion Site-directed Mutagenesis Kit (Thermo Fisher Scientific). The following antibodies were obtained commercially: caspase-3 (1:500 dilution for immunoblotting; Cell Signaling Technology, #9665), cleaved caspase-3 (1:100 dilution for immunoblotting; Cell Signaling Technology, #9661), p62 (1:500 dilution for immunoblotting; Cell Signaling Technology, #5114), Atg3 (1:500 dilution for immunoblotting; Cell Signaling Technology, #3415), Atg4B (1:500 dilution for immunoblotting; Cell Signaling Technology, #5299), Atg5 (11.6 µg/ml for immunofluorescence; Novus Biologicals, NB110-53818), Atg7 (1:500 dilution for immunoblotting; Cell Signaling Technology, #2631), Beclin-1 (1:500 dilution for immunofluorescence; Cell Signaling Technology, #3738), LC3B (1 µg/ml for immunoblotting; Novus Biologicals, NB100-2220), actin (1:2000 dilution for immunoblotting; Sigma, A4700), HA (1 µg/ml for immunofluorescence; Covance, MMS-101P), β-galactosidase (6.25 µg/ml for immunofluorescence; MP Biomedicals, 55976), GluA2 (10 µg/ml for immunofluorescence; Sigma, MAB397). The following reagents were purchased from Sigma: NMDA, APV, rapamycin, and chloroquine. DEVD-fmk (FMK004) and active caspase-3 (707-C3/CF) were purchased from R&D systems. Alexa Fluor^TM^ 555- and Alexa Fluor^TM^ 488-conjugated transferrin was purchased from Thermo Fisher Scientific. The following reagents were purchased from Cayman: trehalose (#20517), bafilomycin A1 (#11038), and leupeptin (#14026). AA2 was purchased from Enzolife Sciences (ALX-420-085). The sequences of primers used to generate DNA constructs are listed in the following table.

**Table:** 

**Plasmid Name**	**Vector**	**Restriction Site**	**Forward (F) and Reverse primers (R)**
EGFP-CA-Rab11	Addgene#56444	Site-directed mutagenesis	**F** – CAGCAGGGCTAGAGCGATATCGAGCTATAACATCAGCATATTATC **R** - GCTCGATATCGCTCTAGCCCTGCTGTGTCCCATATCTGTG
Atg5 siRNA	pSuper	BglII/HindIII	**F** – GGAAGATCTGGCTCACTTTATGTCATGTAAGCTTGGG **R** -CCCAAGCTTACATGACATAAAGTGAGCCAGATCTTCC
Scrambled oligonucleotide	pSuper	BglII/HindIII	**F** – GGAAGATCTGACGTGAACGGATAACACTAAGCTTGGG **R** -CCCAAGCTTAGTGTTATCCGTTCACGTCAGATCTTCC
HA-Atg5	pGW1	XmaI/KpnI	**F** – TCCCCCCGGGATGTGTGGTTTGGACGAATTCCAACTTGTT **R** - CGGGGTACCTGAGCAGCGTAATCTGGAACGTCATATGGAT
siRNA-resistant Atg5	Atg5-siRNA	BglII/HindIII	**F** – GGAAGATCTCGCTCATTTCATGTCATCTAAGCTTGGG **R** - CCCAAGCTTCCCAAGCTTAGATGACATGAAATGAGCG
HA-Beclin-1	pGW1	BglII/EcoRI	**F** – CGCGGATCCATGGAAGGGTCTAAGACGTCCAACAACAG **R** - CCGGAATTCAGCGTAATCTGGAACATCGTATGGGTAGCGG
G2CT	Addgene, #66709	EcoRI/Kpnl	**F**- CCGGAATTCAAGCGGATGAAGCTGAACATCAACCCTAGC **R** – CGGGGTACCGCTAGGGTTGATGTTCAGCTTCATCCGCTT
Scrambled peptide	Addgene, #66709	EcoRI/Kpnl	**F**- CCGGAATTCCGGAAGAACAGCAAGATGCTGATCCCT **R** – CGGGGTACCAGGGATCAGCATCTTGCTGTTCTTCCG
mRFP-LC3	pGW1	KpnI/EcoRI	**F** – CGGGGTACCTCATGGCCTCCTCCGAGGACGTCATC **R** - GCGCGAATTCTCACAAGCATGGCTCTC
mRFP-LC3BG120A	pGW1-mRFP-LC3	Site-directed mutagenesis	**F** – CCCAGGAGACGTTCGCGACAGCACTGGCTGTTACATAC **R** - CCAGTGCTGTCGCGAACGTCTCCTGGGAGGCATAGACC

### Surgery

7-week-old male mice were anesthetized by intraperitoneal injection of Ketamine/Xylazine (Ketamine: 100 mg/kg; Xylazine: 8 mg/kg). A bilateral craniotomy was made above the hippocampus. 200 nl or 1 μl AAV virus was injected into the hippocampal CA1 region (AP: 2.0 mm, ML: ±1.5 mm, DV: −1.5 mm) with a 5 μl gas-tight syringe (Hamilton, #87931, #7803-05) at a speed of 100 nl/min.

### Hippocampal slice culture

6- to 8-day-old male C57BL/6 mice were decapitated, and the brain was placed immediately in cold cutting solution composed of (in mM): 238 sucrose, 2.5 KCl, 26 NaHCO_3_, 1 NaH_2_PO_4_, 5 MgCl_2_, 11 D-glucose and 1 CaCl_2_. Hippocampal slices (350 μm) were cut with a vibratome (Leica, VT1000s) and placed on semi-permeable membrane inserts (Millipore, R7AA72342) in a six-well plate containing culture medium (78.8% minimum essential medium, 20% heat-inactivated horse serum, 25 mM HEPES, 10 mM D-glucose, 26 mM NaHCO_3_, 2 mM CaCl_2_, 2 mM MgSO_4_, 0.0012% ascorbic acid, 1 μg/ml insulin; pH 7.3; 320–330 mOsm). No antibiotics were added and the medium was replaced every 2 days. Slices were biolistically transfected using a gene gun (Bio-Rad, Helios Gene-gun system) at 5–6 days in vitro and used for electrophysiology at 3 days after transfection.

### Acute hippocampal slice

Mice (16–19 days of age, male) were anesthetized with isoflurane and decapitated. The brain was rapidly removed and chilled in ice-cold sucrose solution containing the following (in mM): 2.5 KCl, 26 NaHCO_3_, 1.25 NaH_2_PO_4_, 185 sucrose, 25 D-glucose, 20 HEPES, 5 sodium ascorbate, 2 thiourea, 3 sodium pyruvate, 0.5 CaCl_2_, and 10 MgSO_4,_ pH 7.3. Transverse hippocampal slices (350 μm thick) were cut in ice-cold sucrose solution using a vibratome (VT-1000 s, Leica). The hippocampal slices were incubated in warm (32 °C) ACSF solution (in mM; 124 NaCl, 2.5 KCl, 1.2 NaH_2_PO_4_, 24 NaHCO_3_, 5 HEPES, 12.5 D-glucose, 2 MgCl_2_ and CaCl_2_ pH 7.3) for 30 min and then allowed to cool down to room temperature for 30 min before being transferred to the recording chamber. All solutions were continuously bubbled with 95% O_2_/5% CO_2_.

### Electrophysiology

Hippocampal slices were perfused with ACSF at 2 ml/min. The stimulating electrode was placed on the Schaffer collateral pathway. For field recordings, recording pipettes (1–2 MΩ) were filled with the bath solution and placed in the CA1 region. LTD was induced with low-frequency stimulations (900 pulses, 1 Hz, 15 min), and LTP was induced with high-frequency stimulations (2 trains of 100 pulses at 100 Hz with an inter-train interval of 15 s). For whole-cell recordings, the patch pipette (4–6 MΩ) solution is composed of (in mM): 130.0 cesium methanesulfonate, 8.0 NaCl, 4.0 Mg-ATP, 0.3 Na-GTP, 0.5 EGTA, 10.0 HEPES and 5.0 QX-314 at pH 7.3. EPSCs of CA1 pyramidal neurons were recorded at a holding potential of −70 mV. The series resistance and input resistance were monitored on-line and analyzed with the Clampex program off-line. Only cells with a series resistance of <25 MΩ and a <10% drift in both series resistance and input resistance during the recording period were included.

### Neural culture and transfection

Primary hippocampal neurons were prepared from embryonic day (E) 18–19 rat, male and female embryos. Neurons were seeded on coverslips or culture plates coated with poly-D-lysine (20 μg/ml) and laminin (1.25 μg/ml) at a density of ~750 cells/mm^2^. Neurons were cultured in Neurobasal medium supplemented with 2% B27 and 1% GlutaMax (Thermo Fisher) and transfected with Lipofectamine^TM^ 2000 (Thermo Fisher) following the manufacturer’s instructions.

### GluA2 internalization assay

Neurons (DIV 17) were treated with TTX (1 μM) for 30 min, followed by incubation with an antibody against the N-terminus of GluA2 (10 μg/ml) for 15 min at 37 °C and then NMDA stimulation (30 μΜ, 5 min) or sham treatment. Neurons were fixed with 4% formaldehyde in PBS containing 4% sucrose at 10 min after stimulation. Surface-remaining GluA2 was labeled by incubation with Alexa Fluor® 555-conjugated secondary antibodies (Thermo Fisher). Internalized GluA2 was stained with Alexa Fluor® 488-conjugated secondary antibodies (Thermo Fisher) following permeabilization with methanol (−20 °C, 1 min).

### Transferrin uptake assay

Cultured hippocampal neurons (DIV 17) were washed with warm DMEM (37 °C) for three times before incubation with Alexa Fluor^TM^ 555 (for cells transfected with the constitutively active EGFP-Rab11 plasmid)- or Alexa Fluor^TM^ 488-conjugated transferrin (25 μg/ml in Neurobasal medium) at 37 °C for 15 min Following wash with warm DMEM for three times to remove surface transferrin, neurons were fixed with 4% formaldehyde (in PBS containing 4% sucrose) for imaging acquisition of constitutively active EGFP-Rab11 expressing cells or incubated with the β-galactose antibody (for cells transfected with the β-galactose antibody; 2 h, room temperature) and then with Alexa Fluor® 488-conjugated secondary antibody (1 h, room temperature).

### Immunostaining

Neurons grown on coverslips were fixed with 4% formaldehyde (in PBS containing 4% sucrose) for 15 min at room temperature. Following wash, neurons were incubated with primary antibodies in GDB buffer (12 mM Na_2_HPO_4_, 48 mM NaH_2_PO_4_, 163 mM NaCl, 1% Triton X-100 and 0.36% gelatin at pH 7.4) overnight at 4 °C, washed with PBS, incubated with secondary antibodies in GDB buffer for 1 h at room temperature, washed with PBS, and then mounted on slides with mounting media. For immunohistochemistry in brain sections, P19 mice deeply anesthetized with isoflurane were transcardially perfused with 4% formaldehyde in PBS. Brains were removed, fixed for an additional 12 h with 4% formaldehyde in PBS, and then immersed in 30% sucrose in PBS followed by cutting into 30 μm cryosections with a Leica CM 3050 s cryostat. Brain sections were blocked with 5% horse serum in PBST (PBS with 0.1% Tween 20) followed by incubation with an antibody against Atg5 (Novus, #NB110-53818, 1:500 dilution) overnight at 4 °C. After primary antibody incubation, the sections were washed and incubated with an anti-rabbit, HRP-conjugated secondary antibody (Cell signaling, #7074, 1:500 dilution) at room temperature for 1 h. The sections were washed and developed using TSA plus reagent (PerkinElmer, #NEL741E001KT), then counterstained with DAPI and mounted on slides.

### Image acquisition

For time-lapse imaging, cultured hippocampal slices and primary hippocampal neurons were transferred to a chamber mounted on the sample stage of an Olympus BX61WI confocal microscope, and perfused with artificial cerebrospinal fluid (ACSF) composed of (in mM): 124.0 NaCl, 2.5 KCl, 24.0 NaHCO_3_, 1.2 NaH_2_PO_4_, 2.0 CaCl_2_, 2.0 MgSO_4,_ 12.5 D-glucose, 5.0 HEPES (pH 7.4, bubbled with 95% O_2_/5% CO_2_) at 2 ml/min. Images were acquired with a 60X objective (NA 1.0). Cultured hippocampal slices were stimulated by placing the electrode on the Schaffer collateral pathway near transfected CA1 neurons with low-frequency stimulations (900 pulses, 1 Hz for LTD induction) or high-frequency stimulations (two trains of 100 pulses at 100 Hz with an inter-train interval of 15 s for LTP induction). Images of fixed hippocampal neurons were acquired using a Zeiss LSM780 confocal microscope with a 63X (NA 1.4) objective. Fixed brain sections were imaged on Zeiss LSM 800 confocal microscope with a 10X objective (NA 0.45).

### Image analysis

The time-lapse images were collapsed to make 2D projections using Fluoview 2.1 software. The images of fixed neurons were collapsed to make 2D projections using Zeiss Zen 3.1 software. MetaMorph 6.1 software (Molecular Devices) was used to measure internalized GluA2, surface-remaining GluA2, transferrin, mRFP-LC3, EGFP-Rab5, EGFP-Rab11, LC3 puncta and EGFP-Rab9 on dendrites, and Atg5 and HA in the soma. Briefly, dendrites and soma were traced manually using the Create Region tool. The Threshold Image function was applied to the traced dendrites or soma with the same threshold for all images from one experiment. The Region Measurement tool was used to automatically outline all puncta in the selected area and measure the intensity and size of each puncta. Because previous electron microscopy studies show that the diameter of autophagosomes varies between 300–400 nm and several micrometers^62^, we excluded LC3 positive puncta with diameters <300 nm in the LC3 analysis. To analyze Atg5 immunofluorescence in fixed brain slices, z-stack images were collapsed using Zeiss Zen software, then analyzed with ImageJ software. Somas in the pyramidal cell layer of the hippocampal CA1 and CA3 regions were traced manually using the Polygon Area Selection tool in ImageJ followed by image thresholding, with the same threshold for all images in one experimental group consisting of Cre^−^, CA1Cre^+^ and CA3Cre^+^ brains. Integrated Atg5 immunofluorescence intensity in the pyramidal cell layer was measured using the Particle Analysis function in Image J win-64 software and divided by the number of somas to obtain the mean Atg5 immunofluorescence intensity per cell.

### Behavior

2–4 mice were housed in each cage under a 12 h light/dark cycle at 21–23.3 °C and a relative humidity of 35–60% with food and water ad libitum. 9–12-week-old male littermates were tested for behavior during the dark period. Behavioral data were analyzed using TopScan 3.0 software (CleverSystems). Animal assignment to individual experimental conditions was random.

Open field test. Mice were placed in the center of the test box (49 cm × 49 cm × 40 cm) and allowed to freely explore the arena for 30 min The distance traveled in the whole area and the central area (20 cm × 20 cm) of the box, and the time spent in the central area was analyzed.

Light/dark box test. Mice were placed in the center of the light compartment of a light/dark box (46 cm × 27 cm × 30 cm, two-thirds were illuminated and one third was darkened) and allowed to freely explore the test box for 11 min The time spent in the light and the dark compartment was analyzed.

Elevated O-maze test. The elevated zero maze (60 cm in diameter, Clever Sys Inc.) is divided into four equal sections (two open and two enclosed). Mice were placed in the middle of one open section to begin the test and left on the maze for 6 min The time spent in the closed and the open sections were analyzed.

Fear conditioning. On day 1 of the experiment, the mouse was habituated to the test chamber (Med Associates) for 2 min On day 2, the mouse was exposed to 5 conditioned stimuli (CS; 6000 Hz, 85 dB tone lasting 30 s, with a random inter-stimulus interval of 50–150 s), each co-terminated with an unconditioned stimulus (US; electrical foot-shock, 0.5 s, 0.75 mA). Contextual fear memory was tested at 1 and 24 h after fear conditioning by placing the mouse in the fear conditioning context for 6 min Cued fear memory was tested at 24 h after fear conditioning by exposing the mouse to a tone (6000 Hz, 85 dB, lasting 2 min) in a context different from the fear conditioning context. Freezing was identified by complete immobility (except for respiratory movement) with the Video Freeze 2.15 software (Med Associates).

### Statistical analysis

The SigmaPlot 13.0 and IBM SPSS Statistics 26 software was used for statistical analysis. For the comparison of two groups, data were analyzed by two-tailed Student’s *t* test or paired Student’s *t* test. To test for differences among more than two groups, normally distributed data with equal variance were analyzed using one-way ANOVA, one-way RM ANOVA or two-way ANOVA. The data that did not pass the normality and equal variance tests were analyzed using Kruskal–Wallis one-way analysis of variance on ranks. Student–Newman–Keuls test, Bonferroni test, Holm–Sidak test or Dunn’s test were used for post hoc analysis to identify significantly different groups. All statistical analysis was two-tailed and *p* < 0.05 was considered significant. The specific statistical analysis method for each experiment is described in Figure legends. Sample size was estimated from similar experiments previously done in the lab. No data were excluded from the analysis. All results were produced from ≥2 independent experiments and replicated. All animal assignment and data analyses were done blindly to the experimental condition.

### Reporting summary

Further information on research design is available in the Nature Research Reporting Summary linked to this article.

## Supplementary information

Supplementary Information

Reporting Summary

## Data Availability

The data that support the findings of this study are provided as a Source Data file. Uncropped immunoblots were provided in Supplementary Fig. 9. Source data are provided with this paper.

## Source data

Source Data

## Code availability

No code is generated by this study.
