# Supplementary material for: Autophagy controls the induction and developmental decline of NMDAR-LTD through endocytic recycling
Source: Nat Commun. 2020 Jun 12;11:2979. doi: 10.1038/s41467-020-16794-5 (PMC7293213; doi:10.1038/s41467-020-16794-5)

Supplementary Information

# Autophagy controls the induction and developmental decline of NMDAR-LTD through endocytic recycling

Hongmei Shen, Huiwen Zhu, Debabrata Panja, Qinhua Gu, Zheng Li

# Supplementary Figure 1

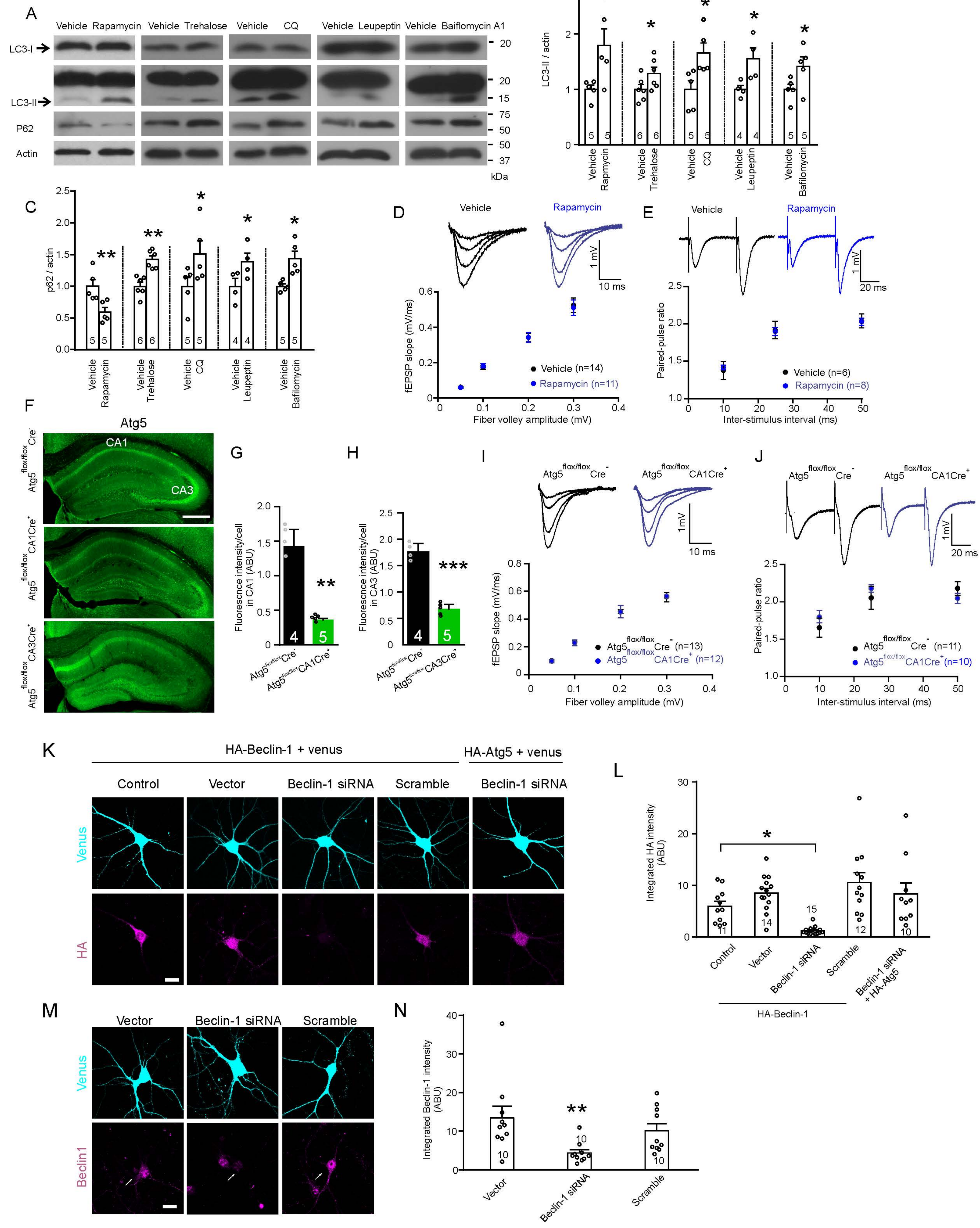

**Supplementary Figure 1. The effect of autophagy activators and autophagy inhibitors on autophagy in hippocampal slices, Atg5 expression in conditional Atg5 knockout mice, the effect of rapamycin and CA1-specific Atg5 knockout on basal synaptic transmission, and the specificity and efficacy of Beclin-1 siRNAs.** (A) Representative blots of CA1 lysates (17–19–day old, wild-type). (B, C) Quantification for A (vehicle vs. rapamycin: $p = 0.0384$; vehicle vs. trehalose: $p = 0.031$; vehicle vs. CQ: $p = 0.0165$; vehicle vs. leupeptin: $p = 0.035$; vehicle vs. bafilomycin A1: $p = 0.035$ for B; vehicle vs. rapamycin: $p = 0.00137$; vehicle vs. trehalose: $p = 0.00192$; vehicle vs. CQ: $p = 0.0432$; vehicle vs. leupeptin: $p = 0.0241$; vehicle vs. bafilomycin A1: $p = 0.0169$ for C); two-tailed paired t-test or Wilcoxon Signed Rank Test was used for statistical analysis; n in the bars indicate the number of animals. (D, E, I, J) Input-output relationship and paired-pulse ratio in 17–19-day old wild-type hippocampal slices; n indicates the number of slices from 3 animals. Rapamycin (1 μM) was added to the bath at 30 min before recording. (F) Representative images for Atg5 staining for mice at 19 days of age; scale bar (500 μm). (G, H) Fluorescence intensity of Atg5 per cell; two-tailed Student's t-test was used for statistical analysis and n in the bar indicates the number of slices from 3 animals ($p = 0.0016$ for G, $p = 0.0004$ for H). (K, M) Representative images of cultured hippocampal neurons; scale bar, 20 μm. (L) Quantification for K; Kruskal-Wallis one-way ANOVA on ranks was used to compare across groups ($p = 5.1471 \times 10^{-7}$), and Dunn's test was used to identify groups significantly different from the control group. (N) Quantification for M; Kruskal-Wallis one-way ANOVA on ranks was used for statistical analysis ($p = 0.004$), and Dunn's test was used to identify groups significantly different from the vector group; the number in the bar indicates the number of cells. Data are presented as mean ± SEM; * $p < 0.05$, ** $p < 0.01$, *** $p < 0.001$; no adjustments were made for multiple comparisons; ABU: arbitrary unit.

# Supplementary Figure 2

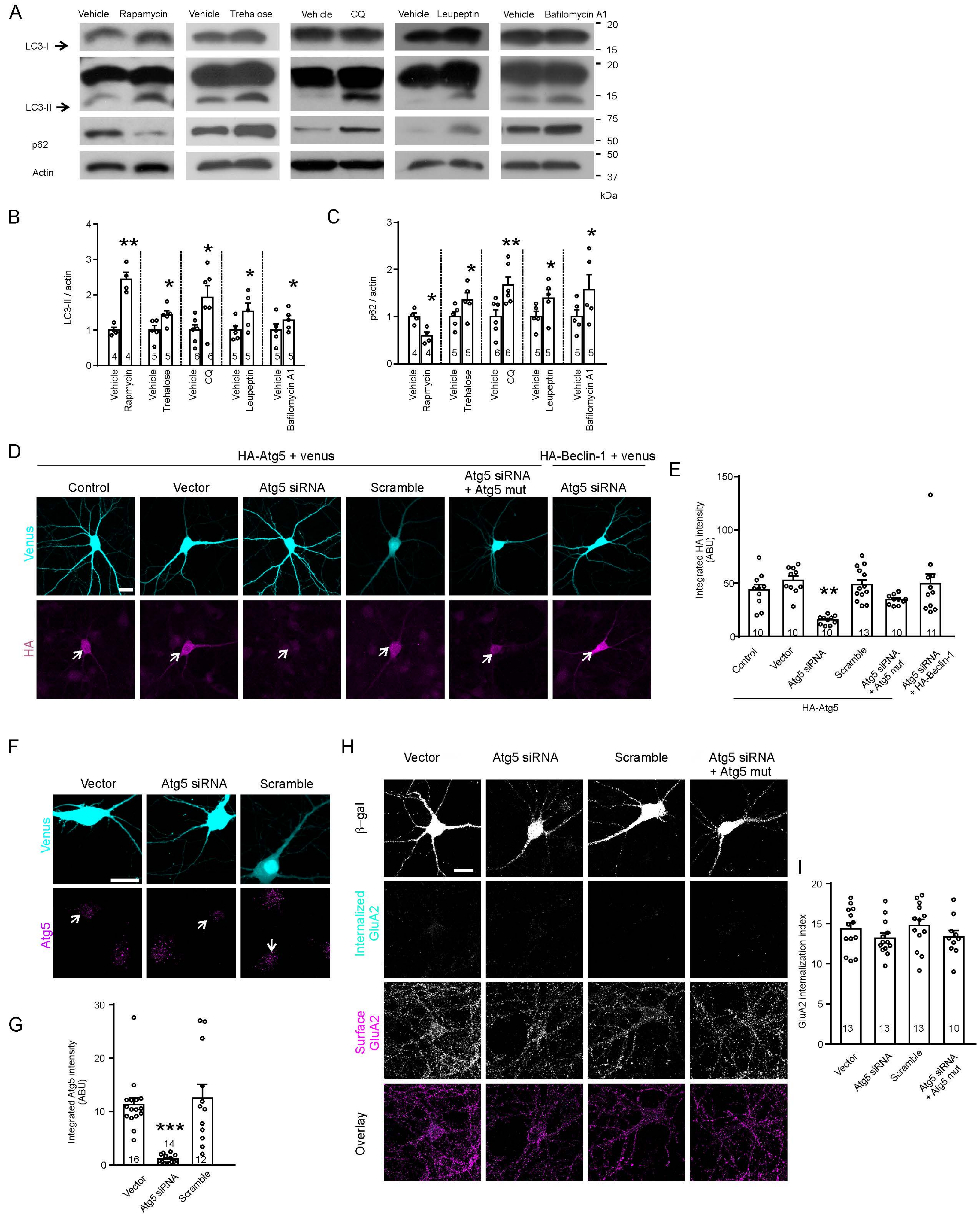

**Supplementary Figure 2. The effect of autophagy activators and inhibitors on autophagy in primary hippocampal neurons, and Atg5 siRNA's efficacy, specificity, and effect on basal GluA2 internalization.** (A) Representative blots of primary hippocampal neurons treated with rapamycin (1 μM), trehalose (20 mM), CQ (120 μM), leupeptin (300 μM), bafilomycin A1 (20 μM) or vehicle. (B, C) Quantitation for A (vehicle vs. rapamycin: p = 0.00126; vehicle vs. trehalose: p = 0.0245; vehicle vs. CQ: p = 0.0312; vehicle vs. leupeptin: p = 0.0163; vehicle vs. bafilomycin A1: p = 0.0155 for B; vehicle vs. rapamycin: p = 0.0463; vehicle vs. trehalose: p = 0.0385; vehicle vs. CQ: p = 0.00207; vehicle vs. leupeptin: p = 0.0127; vehicle vs. bafilomycin A1: p = 0.0384 for C), two-tailed paired t-test was used for statistical analysis. (D, F, H) Representative images of primary hippocampal neurons; scale bar, 20 μm. (E) Quantitation for D; Kruskal-Wallis one-way ANOVA on ranks was used to compare across groups (p = 0.000006), and Dunn's test was used to identify groups significantly different from the control group. (G) Quantification for F; Kruskal-Wallis one-way ANOVA on ranks was used to compare across groups (p = $2 \times 10^{-6}$), and Dunn's test was used to identify groups significantly different from the vector group. (I) Quantification for H. Data are presented as mean ± SEM; the number in the bar indicates the number of cells in each group; * $p < 0.05$, ** $p < 0.01$, *** $p < 0.001$; no adjustments were made for multiple comparisons; ABU: arbitrary unit.

Supplementary Figure 3

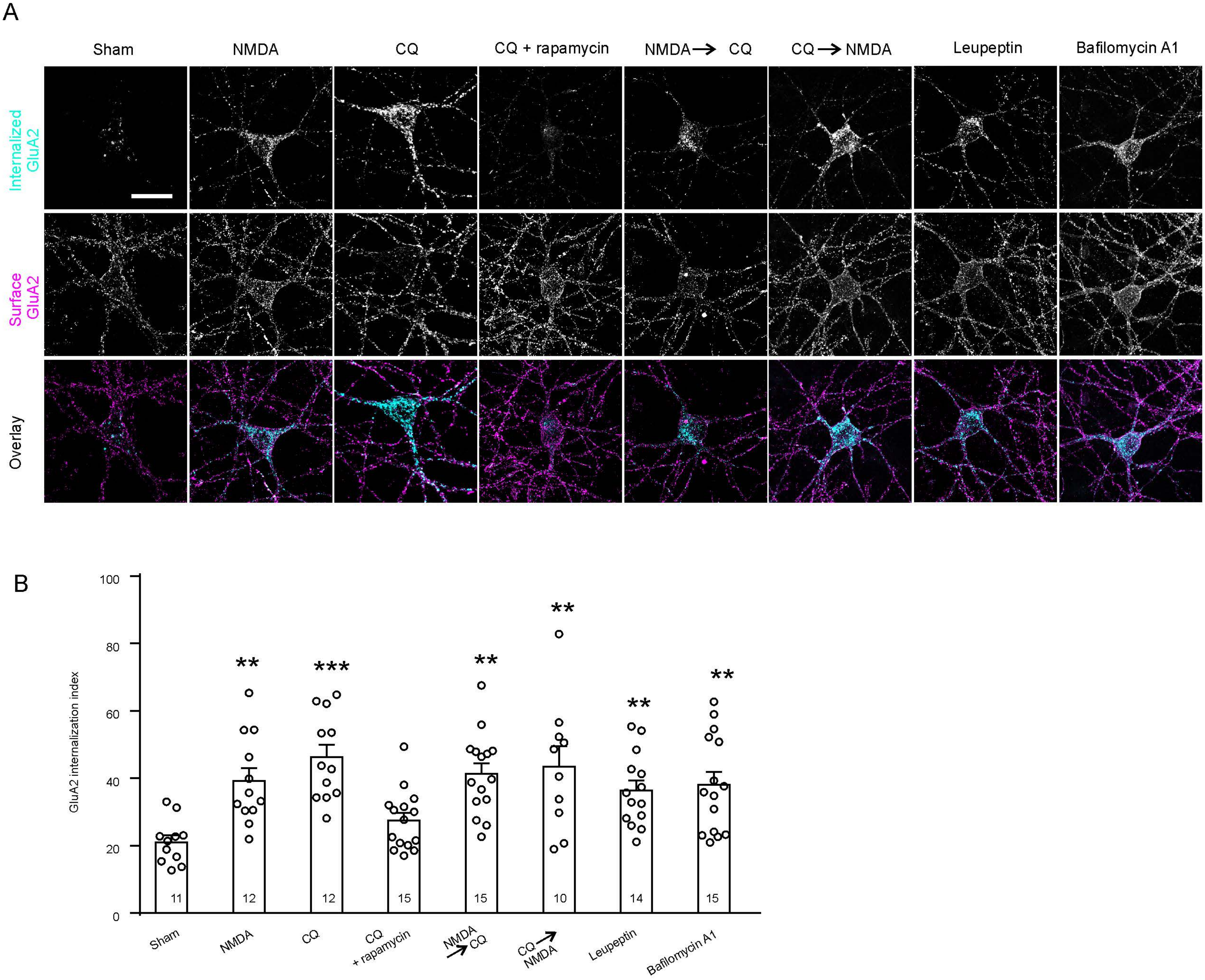


**Supplementary Figure 3. Autophagy inhibition promotes AMPA receptor internalization.** (A) Representative images of primary hippocampal neurons treated with NMDA (30 μM, 5 min), CQ (120 μM, 10 min), leupeptin (300 μM, 30 min), and bafilomycin A1 (20 μM, 30 min) alone or in combination; scale bar, 20 μm. (B) Quantification for A; one-way ANOVA was used to compare across groups ($p = 0.000028$); groups significantly different from the sham group were identified with Student-Newman-Keuls test and marked with asterisks. The graph shows mean ± SEM; the number in the bar indicates the number of cells; $^{**}p < 0.01$, $^{***}p < 0.001$; no adjustments were made for multiple comparisons.

# Supplementary Figure 4

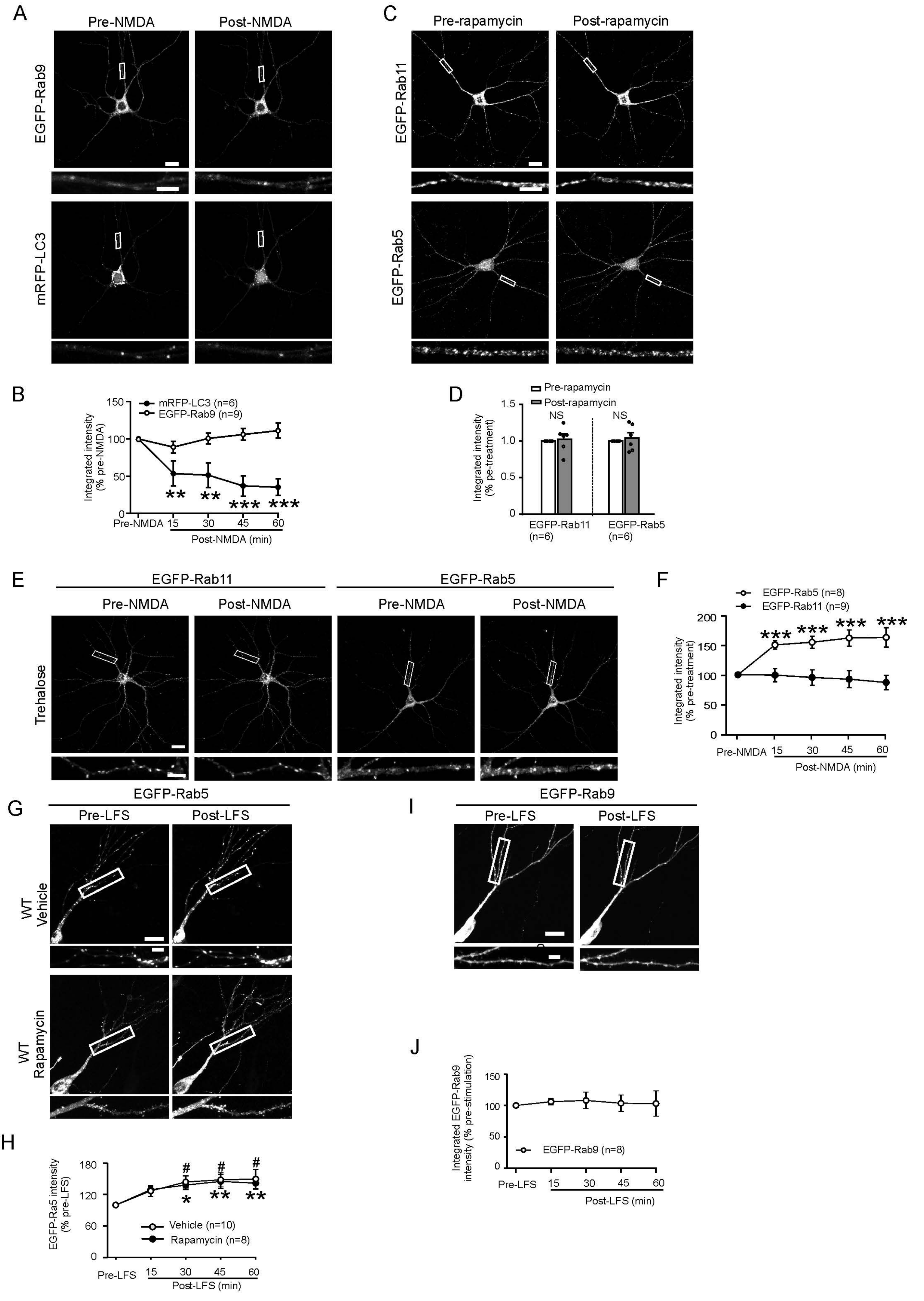

**Supplementary Figure 4. Late Endosomes are not significantly altered in LTD, rapamycin has no effect on recycling or early endosomes, and trehalose blocks the reduction of recycling endosomes in LTD.** (A) Representative images of primary hippocampal neurons before and after NMDA (30 μM, 5 min) treatment. (B) Quantification for A; one-way RM ANOVA was used to compare across time points ($p = 0{,}00026$ for mRFP-LC3, $p = 0.93$ for EGFP-Rab9); Holm-Sidak test was used to identify time points significantly different from the pre-stimulation baseline; *** $p < 0.001$. (C) Representative images of neurons before and after rapamycin treatment (1 μM, 30 min). (D) Quantification of EGFP-Rab11 and EGFP-Rab5 in C; two-tailed paired t-test was used for statistical analysis ($p = 0.704$ for EGFP-Rab11, $p = 0.576$ for EGFP-Rab5). (E) Representative images of neurons before and after treatment with NMDA (30 μM, 5 min) along with trehalose (20 mM, pretreated for 30 min and present throughout the experimental period). (F) Quantification of EGFP-Rab11 and EGFP-Rab5 in E; one-way RM ANOVA was used to compare across time points ($p = 3 \times 10^{-6}$ for EGFP-Rab5, $p = 0.756$ for EGFP-Rab11); Holm-Sidak test was used to identify time points significantly different from the pre-stimulation baseline; *** $p < 0.001$. (G, I) Representative images of the same dendrites of neurons in wild-type hippocampal slices before and after low-frequency stimulation (LFS). (H) Quantification of EGFP-Rab5 in G; one-way RM ANOVA was used to compare across time points; WT + vehicle: $p = 0.001$; WT + rapamycin: $p = 0.008$; Holm-Sidak test was used to identify time points significantly different from the pre-stimulation baseline. (J) Quantification of EGFP-Rab9 in I; one-way RM ANOVA was used to compare across time ($p = 0.97$); * $p < 0.05$, ** $p < 0.01$ for the vehicle group; $^{\#}$ $p < 0.05$ for the rapamycin group; no adjustments were made for multiple comparisons. Data are presented as mean ± SEM; n indicates the number of cells in each group. Scale bar: 20 μm in top images, 5 μm in lower images.

Supplementary Figure 5

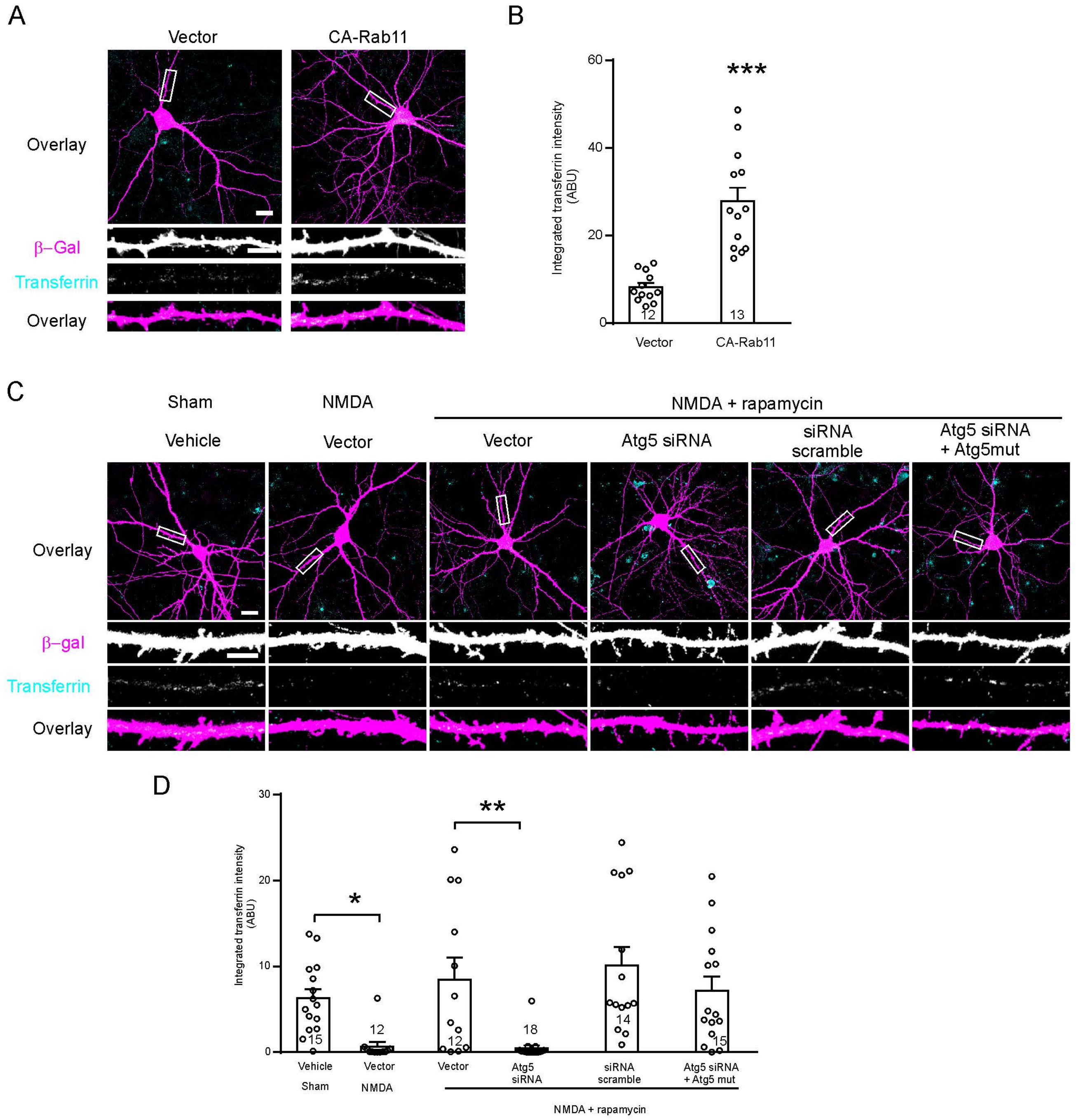


**Supplementary Figure 5. Autophagy inhibition is required for reduced transferrin uptake in LTD.** (A, C) Representative images of transferrin uptake in primary hippocampal neurons incubated with the Alexa FluorTM 488-conjugated transferrin for 15 min. (B) Quantification for A; Mann-Whitney Rank Sum Test was used for statistical analysis ($p = 0.000022$). (D) Quantitation for C; Kruskal-Wallis one-way ANOVA on ranks was used to compare across groups ($p = 4.89 \times 10^{-8}$), and Dunn's test was used for post hoc analysis (no adjustments were made for multiple comparisons); * $p < 0.05$, ** $p < 0.01$, *** $p < 0.001$. Data are shown as mean ± SEM; n indicates the number of cells in each group; scale bars, 20 μm in upper images and 5 μm in lower, enlarged images. ABU, arbitrary unit.

# Supplementary Figure. 6

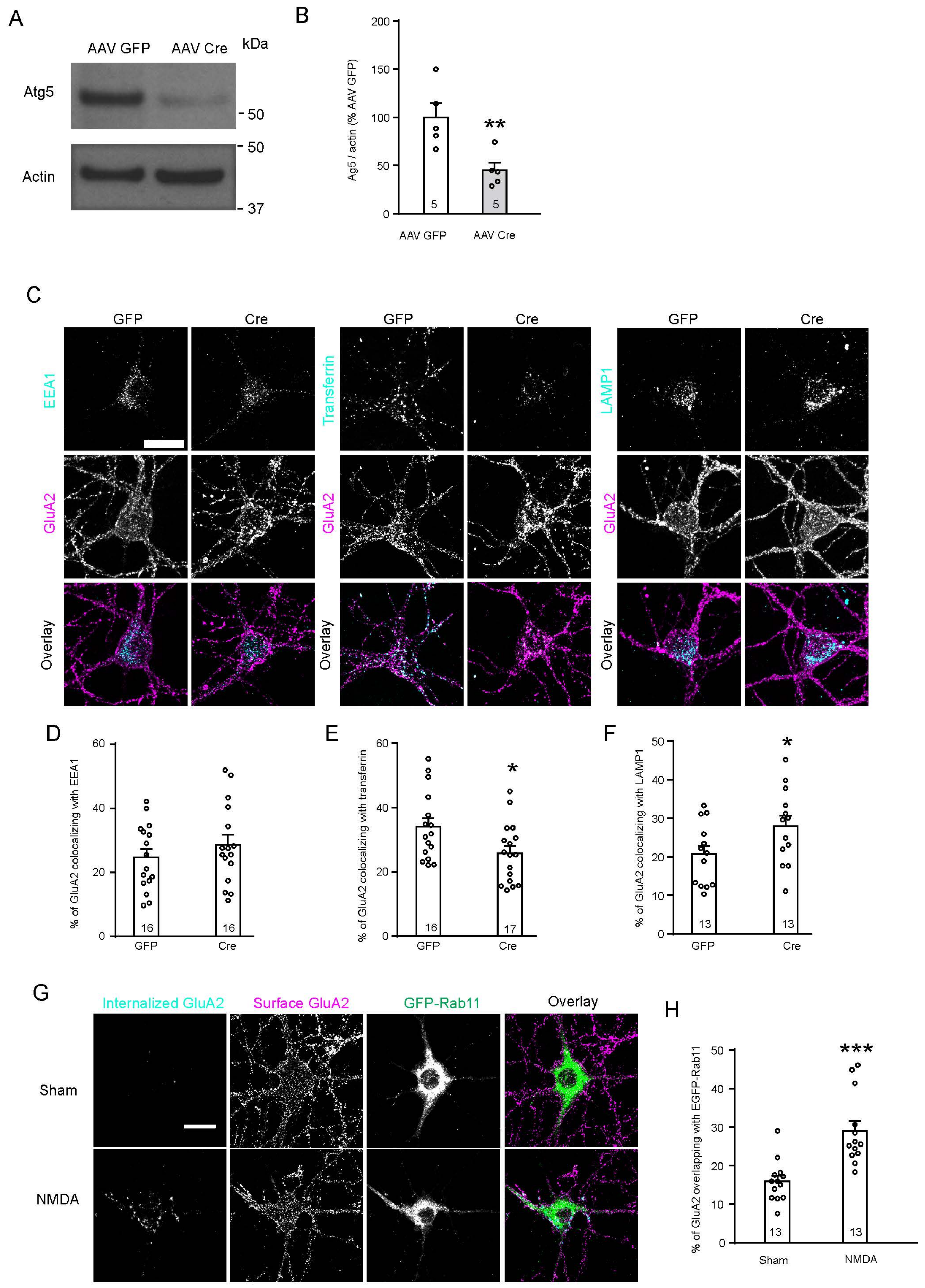

**Supplementary Figure 6. Endocytic trafficking of GluA2 in Atg5 knockout cells.** (A) Representative blots of primary hippocampal neurons transduced with AAV at DIV 4 and lysed at DIV 17. (B) Quantification for A; two-tailed paired t-test was used for statistical analysis ($p = 0.00424$). (C) Representative images of primary hippocampal neurons transduced with AAV Cre or AAV GFP at DIV 4 and incubated with the GluA2 antibody alone or along with transferrin for 15 min, then fixed for immunostaining; scale bar, 10 μm. (D, E, F) Quantitation for A; two-tailed Student's t-test was used in D and F, and Mann-Whitney Rank Sum Test was used in E ($p = 0.346$ for D, $p = 0.029$ for E, $p = 0.0483$ for F). (G) Representative images of GluA2 internalization in primary hippocampal neurons; scale bar, 20 μm. (H) Quantification of the percentage of GluA2 colocalized with Rab11 in G; Mann-Whitney Rank Sum Test was used for statistical analysis ($p = 0.000164$); scale bar, 20 μm. Data are presented as mean ± SEM; * $p < 0.05$, ** $p < 0.01$; n in the bar indicates animal number in B and cell number in D–F and H.

# Supplementary Figure 7

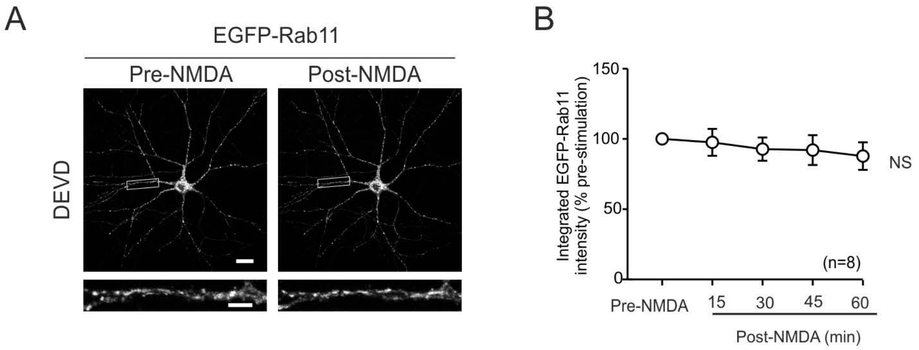


**Supplementary Figure 7. Recycling endosomes are unchanged after NMDA stimulation in the presence of a caspase-3 inhibitor.** (A) Representative images of primary hippocampal neurons before and after NMDA treatment (30 μM, 5 min); DEVD (5 μM) was added to the medium and pretreated for 30 min.
(B) Quantification for A; one-way RM ANOVA was used to compare across time points ($p = 0.684$). Scale bar: 20 μm in upper images and 5 μm in lower images. Data are presented as mean ± SEM; n indicates the cell number.

Supplementary Figure 8

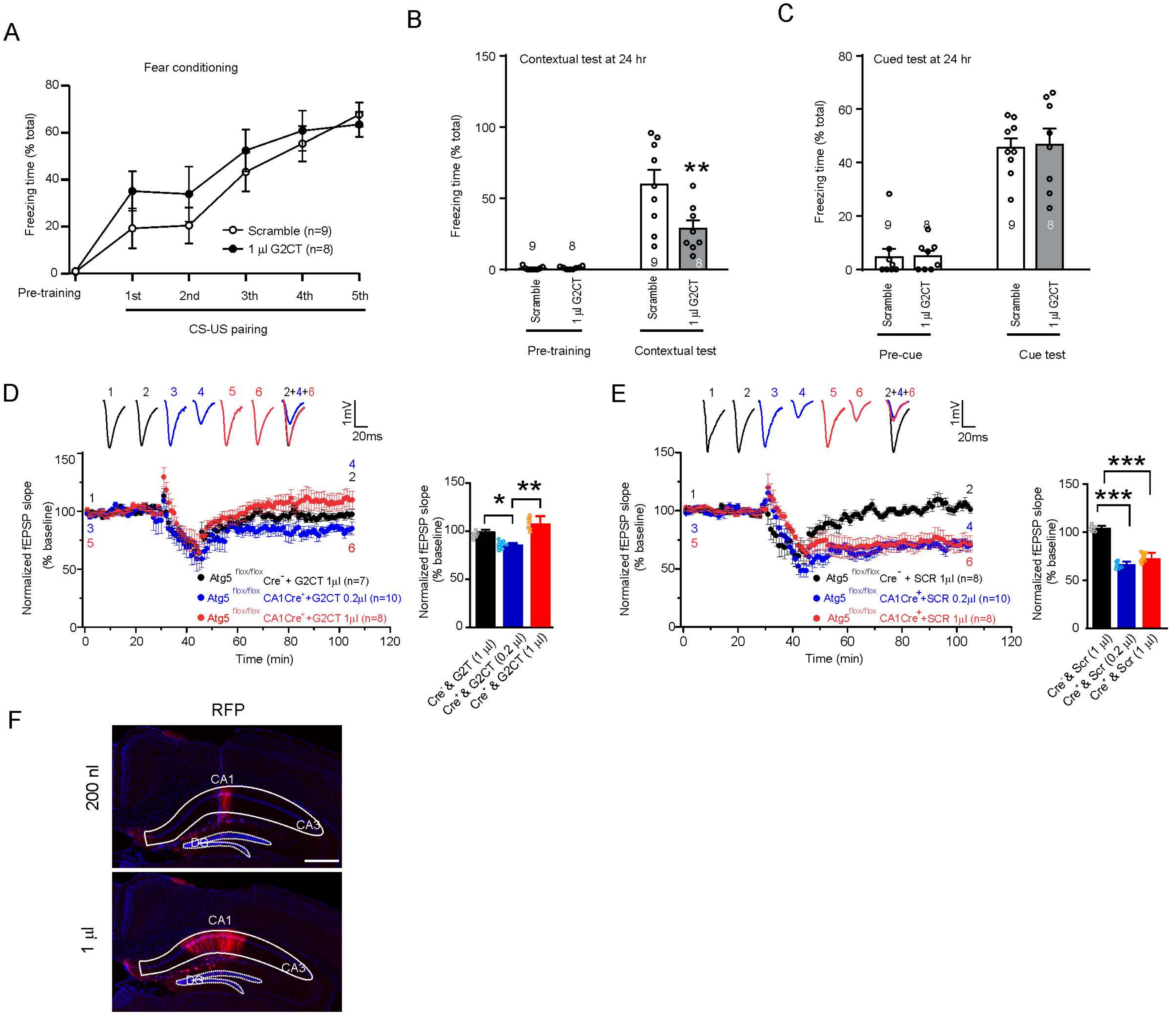


**Supplementary Figure 8. Contextual fear memory is impaired by injection of 1 μl G2CT virus into the hippocampal CA1 region.** (A–C) Atg5$^{flox/flox}$Cre$^{-}$ (8 weeks of age) mice were injected with 1 μl AAV expressing G2CT or scramble peptide and tested for contextual fear memory 4 weeks later. (A) The percentage of time spent in freezing before and after each CS-US pairing. (B) Contextual fear memory tested at 24 hr after fear conditioning (two-way ANOVA was used for statistical analysis; p = 0.014 for interaction between the effects of genotype and test on freezing; p = 0.001 for the simple main effect of scramble vs. G2CT within the contextual test). (C) Cued fear memory tested at 24 hr after fear conditioning. (D, E) LTD in the CA1 region was induced by LFS in hippocampal slices injected with AAV expressing G2CT or scramble peptide along with red fluorescent protein (RFP); quantification on the right shows fEPSPs recorded at 50–60 min after LFS normalized to the pre-stimulation baseline; one-way ANOVA was used for comparison across groups (p = 0.002 for D and p = $3.7 \times 10^{-11}$ for E); Bonferroni test was used for post hoc analysis (no adjustments were made for multiple comparisons). Data are presented as mean ± SEM; n and the number in the bar indicates the number of animals (one slice per animal); * p < 0.05, ** p < 0.01, *** p < 0.001. (F) Representative images from 4 animals injected with 200 nl and 4 animals injected with 1 μl of AAV expressing G2CT along with RFP; scale bar, 500 μm.

# Supplementary Figure 9. Uncropped image of Western blots

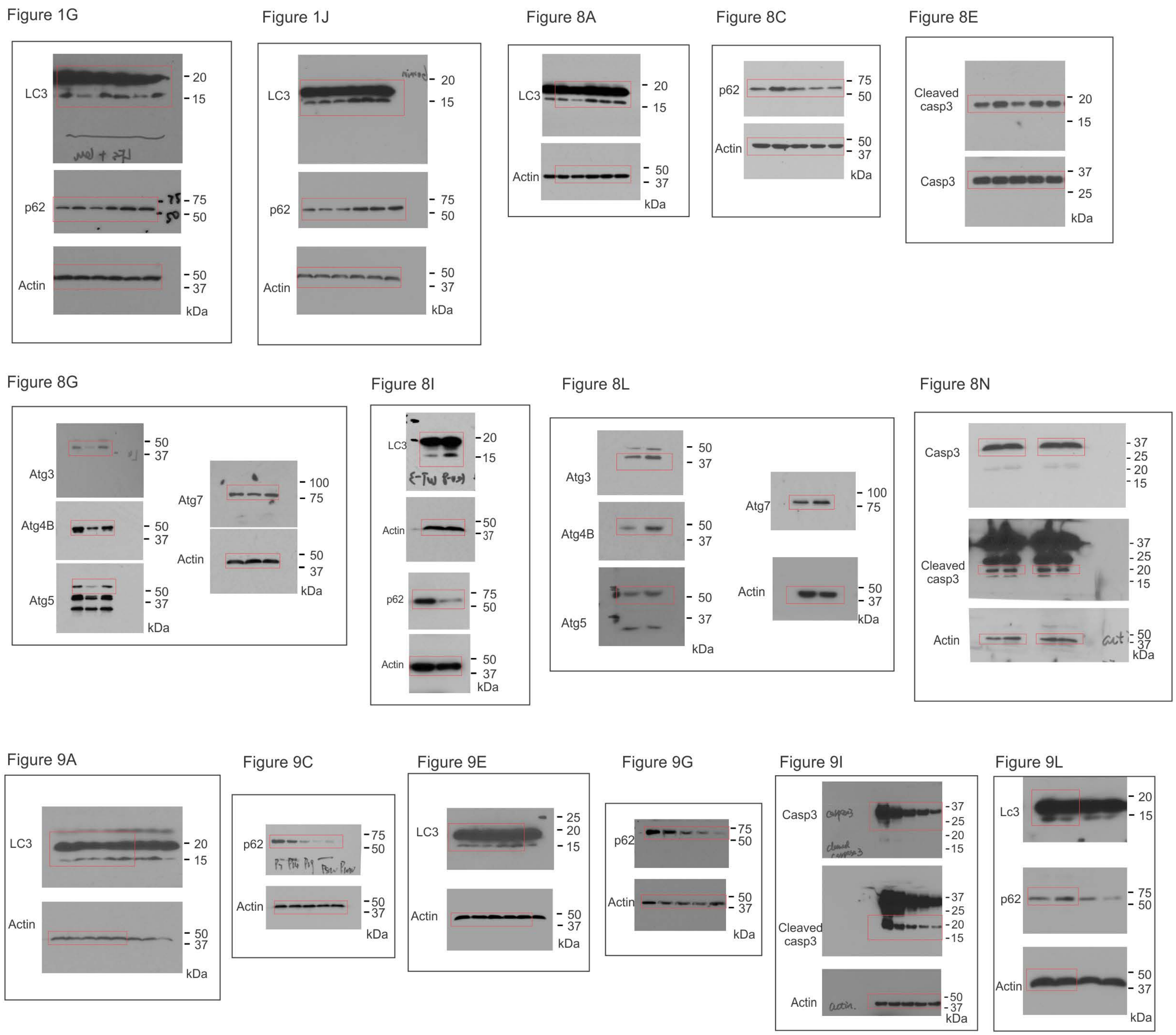

## Supplementary Figure 9 (continued)

Supplementary Fig. 1A

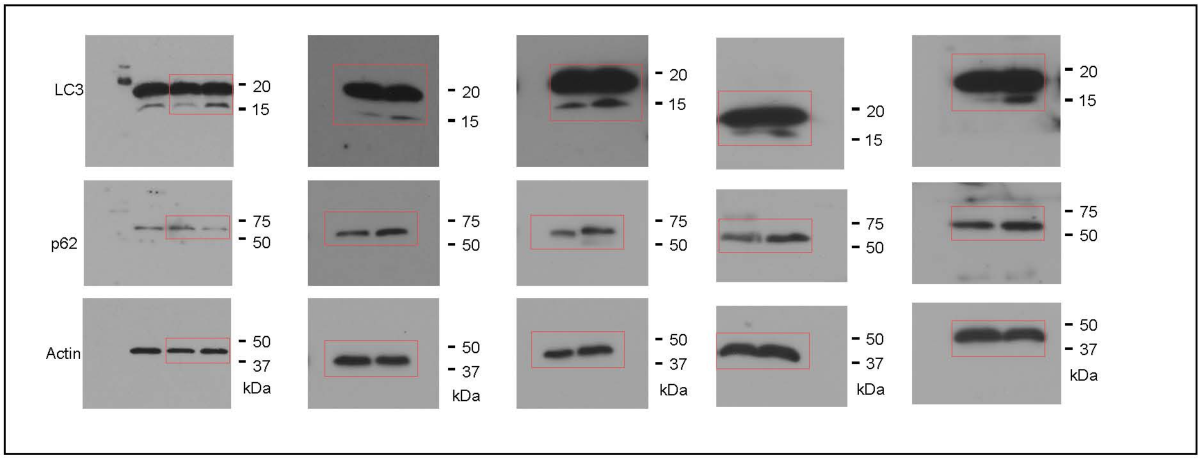


Supplementary Fig. 2A

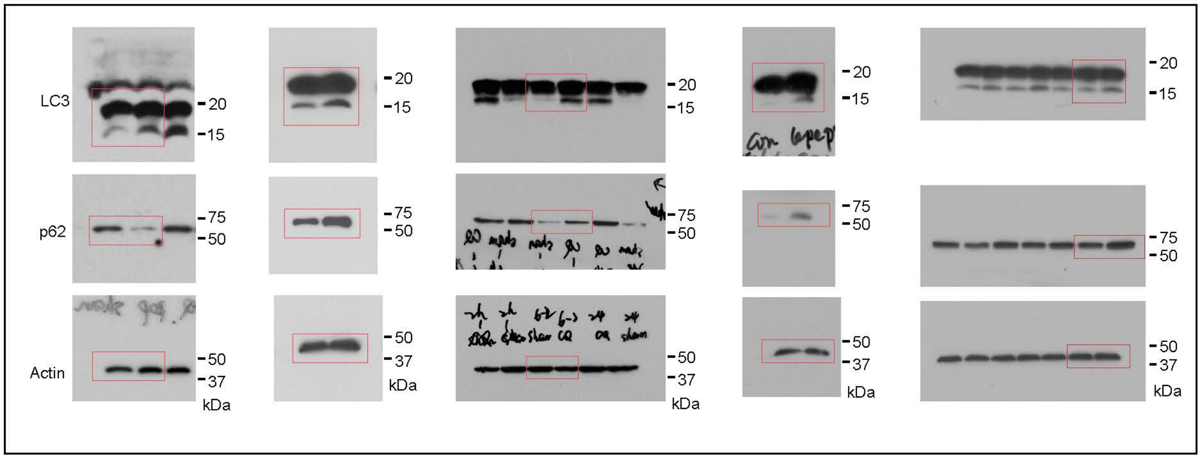


Supplementary Fig. 6A

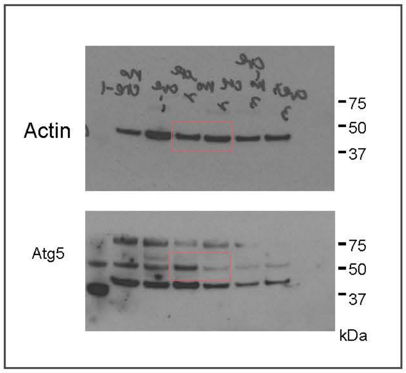

Supplement: Supplementary file 1 — Supplementary Information [file 41467_2020_16794_MOESM1_ESM.pdf]
